# Supplementary material for: Evidence of dengue transmission and a diverse Aedes mosquito virome on the Congo’s Angola border
Source: Nat Commun. 2026 Aug 10;17:9560. doi: 10.1038/s41467-026-76453-z (PMC13550466; doi:10.1038/s41467-026-76453-z)
Supplement: Supplementary file 1 [file 41467_2026_76453_MOESM1_ESM.pdf]

Evidence of dengue transmission and a diverse *Aedes* mosquito virome on the Congo's Angola border

Running title: *Aedes* mosquito virome in the DRC

Wenqiao He[1#], Thierry Bobanga[2], Anne Piantadosi[3,4], Zachary R. Popkin-Hall[1,5], Fabien Vulu[2], Matthew H. Collins[4], Melchior M. Kashamuka[6], Antoinette K. Tshefu[6], Jonathan J. Juliano[1*], Jonathan B. Parr[1*#]

[1] Institute for Global Health and Infectious Diseases and Division of Infectious Diseases, Department of Medicine, University of North Carolina at Chapel Hill, Chapel Hill, NC, United States

[2] Department of Tropical Medicine, Faculty of Medicine, University of Kinshasa, Kinshasa, Democratic Republic of the Congo

[3] Department of Pathology and Laboratory Medicine, Emory University School of Medicine, Atlanta, GA, USA

[4] Division of Infectious Diseases, Department of Medicine, Emory University School of Medicine, Atlanta, GA, USA

[5] Department of Biology, Western Connecticut State University, Danbury, CT, USA

[6] Kinshasa School of Public Health, Kinshasa, Democratic Republic of the Congo

* These authors jointly supervised this work.

[#] Corresponding authors: wenqiao@email.unc.edu; jonathan_parr@med.unc.edu

SUPPLEMENTARY NOTES

The main manuscript presents results from metagenomic analysis conducted after applying conservative filters implemented to reduce the risk of false-positive calls. Here, we include additional results of this analysis at the viral family level, as well as results of a separate analysis conducted without the same conservative filters. We have included the latter results because this more lenient approach enables detection of lower frequency viruses within the pools. However, results should be interpreted carefully, with knowledge that there is increased risk of false-positive calls.

Viral metagenomic analysis at the family level - additional detail (after applying the 10 RPM threshold and an index-hopping filter)

A total of four viral families—*Iflaviridae*, *Sedoreoviridae*, *Narnaviridae,* and *Totiviridae*—are consistently detected in mosquito pools across all sampling sites. Among these, genera from three families are also present across all sampling sites, including *Iflavirus* (*Iflaviridae*), *Orbivirus* (*Sedoreoviridae*), and *Totivirus* (*Totiviridae*) (Figure 4). Reads mapping to *Baculoviridae*, *Alphatetraviridae*, *Mesoniviridae*, and *Permutotetraviridae* are detected in the field mosquito pool collected from Malanga, while none of the pools collected from Kimpese city or Viaza are positive for these viruses. All viral families detected in mosquitoes from Viaza are also present in samples from Kimpese city. *Solemoviridae* exhibit the highest relative abundance in most mosquito pools collected from Kimpese city and Viaza (except for pool K2), while *Permutotetraviridae* is the dominant viral family in mosquitoes from Malanga (Figure 4 and Supplementary Fig. 2). The majority of reads within *Solemoviridae* and *Permutotetraviridae* are annotated as insect-specific viruses. Notably, a high relative abundance of *Picornaviridae* and *Dicistroviridae* is observed in one mosquito pool from Kimpese city (K2), with most reads within these families also classified as insect-specific viruses. A relatively high abundance of reads mapping to the *Flaviviridae* family is also observed, except in mosquitoes collected from Malanga and the K2 pool from Kimpese city. Viral families known to include human and animal pathogens and potential pathogens, such as *Flaviviridae, Picornaviridae*, *Rhabdoviridae*, and *Orthomyxoviridae,* are detected.

Viral metagenomic analysis at the family level - additional detail (prior to applying the 10 RPM threshold and index-hopping filter)

Based on alignment of the NCBI-nt database, we identify 51 unique viral families in the field mosquito pools, ranging from 7-29 viral families per pool. A total of 17 shared viral families are identified in mosquitoes across all three sample collection areas, with reads mapping to *Solemoviridae* and *Flaviviridae* detected in all field mosquito pools. Similar to the results after applying the RPM <10 threshold and index-hopping filter, the viral family *Solemoviridae* has the highest relative abundance in most of the field mosquito pools collected in Kimpese city and Viaza, while *Permutotetraviridae* has the highest relative abundance in mosquitoes collected in Malanga (Supplementary Fig. 8a). Except for the mosquito pool from Malanga, *Flaviviridae* is among the top three viral families in other mosquito pools. High relative abundance of *Picornaviridae* and *Dicistroviridae* is observed in one mosquito pool collected in the Kimpese city (K2).

Viral metagenomic analysis at the genus level - additional detail (prior to applying the 10 RPM threshold and index-hopping filter)

We observe a range of 5 to 24 viral genera per field mosquito pool, comprising 64 unique total viral genera. Six viral genera are present in field mosquito pools collected from all sample collection areas, with *Orthoflavivirus* found in all field mosquito pools tested. Similar to the filtered results, *Orthoflavivirus* is the most abundant viral genus in most mosquito pools collected from Kimpese city and Viaza, while the insect virus *Jonvirus* is the genus with the highest relative abundance in the mosquito pool collected in Malanga (Supplementary Fig. 8b). PCA based on viral genus annotation to explore clustering by sample collection area is shown in Supplementary Fig. 9. Potential geographical clustering is observed among samples from Kimpese city and Viaza. The Malanga mosquito pool is distinct from other samples.

Phylogenetic analysis of reads mapping to potential pathogens - additional detail (prior to applying the 10 RPM threshold and index-hopping filter)

BLAST-confirmed reads mapping to viral species related to human or animal diseases, including *human pegivirus* and *human blood-associated dicistrovirus*, are observed in the mosquito pools prior to applying the 10 RPM threshold and index-hopping filter for viral metagenomic data analysis. One consensus sequence showed high similarity with a published human pegivirus *polyprotein* gene sequence (MZ099567.1, 7917-8114, 228bp) from Thailand was found in a mosquito pool from Kimpese city (Supplementary Fig. 10). *Human blood-associated dicistrovirus* reads are detected in two mosquito pools from the Kimpese city. The consensus

sequences generated from those reads show high similarity with the regions of the structural polyprotein (K4 and K5 reads), as well as the non-structural polyprotein (K5 read) of an isolate from Cameroon (OQ835731.1, 8846-8978, 133bp; 9276-9430, 155bp; 5259-5406, 148bp) (Supplementary Fig. 11).

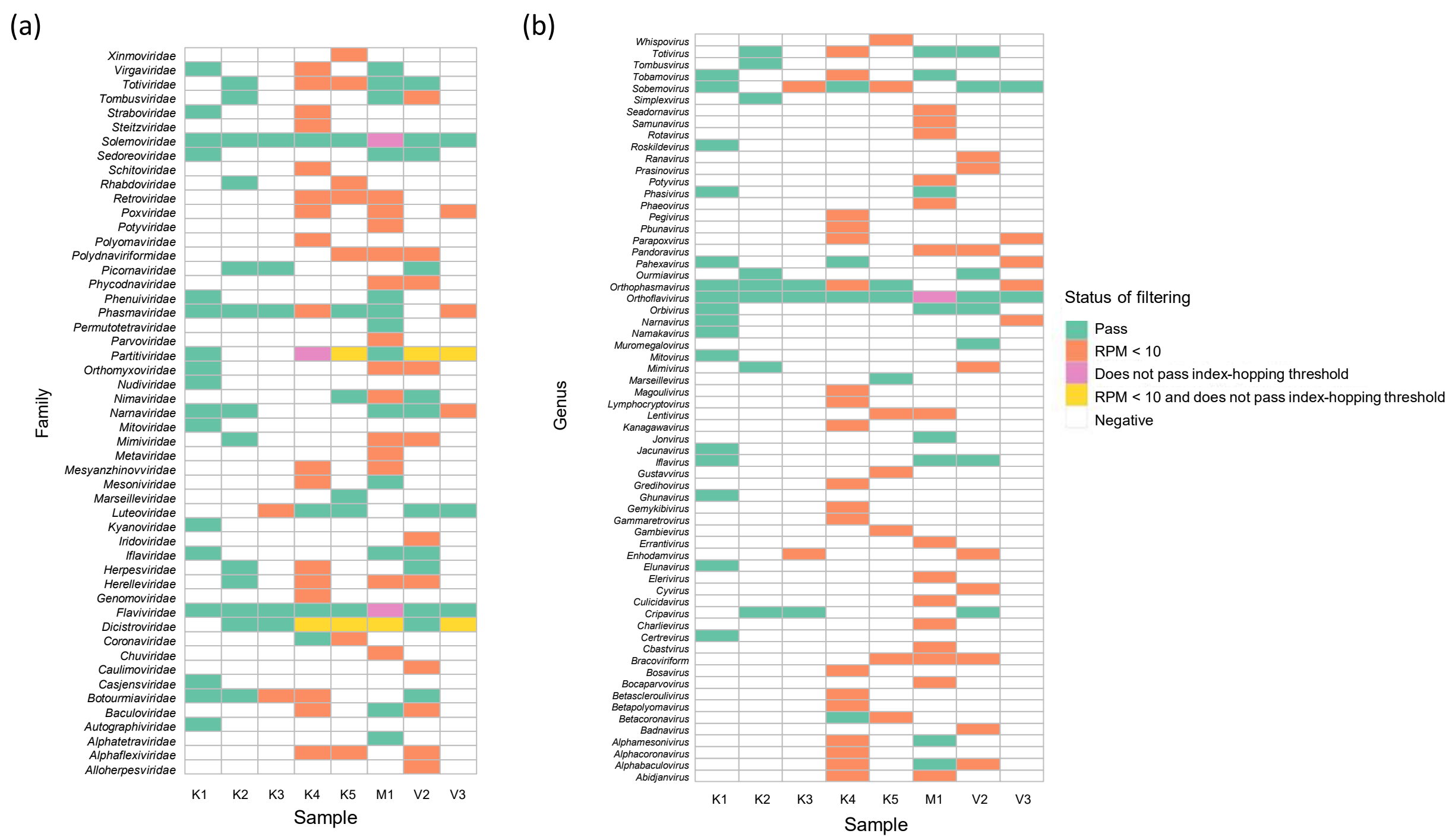

Supplementary Fig. 1 Filtering results by viral family and genus. After applying the reads per million viral reads (RPM) < 10 threshold and index-hopping filter, a) a total of 33 unique total viral families are detected, with each pool containing between 3-17 passed viral families, and b) a total of 28 unique total viral genera are detected, with each pool containing between 2-16 passed viral genera. Shaded cells have detected reads, with color denoting filtering result.

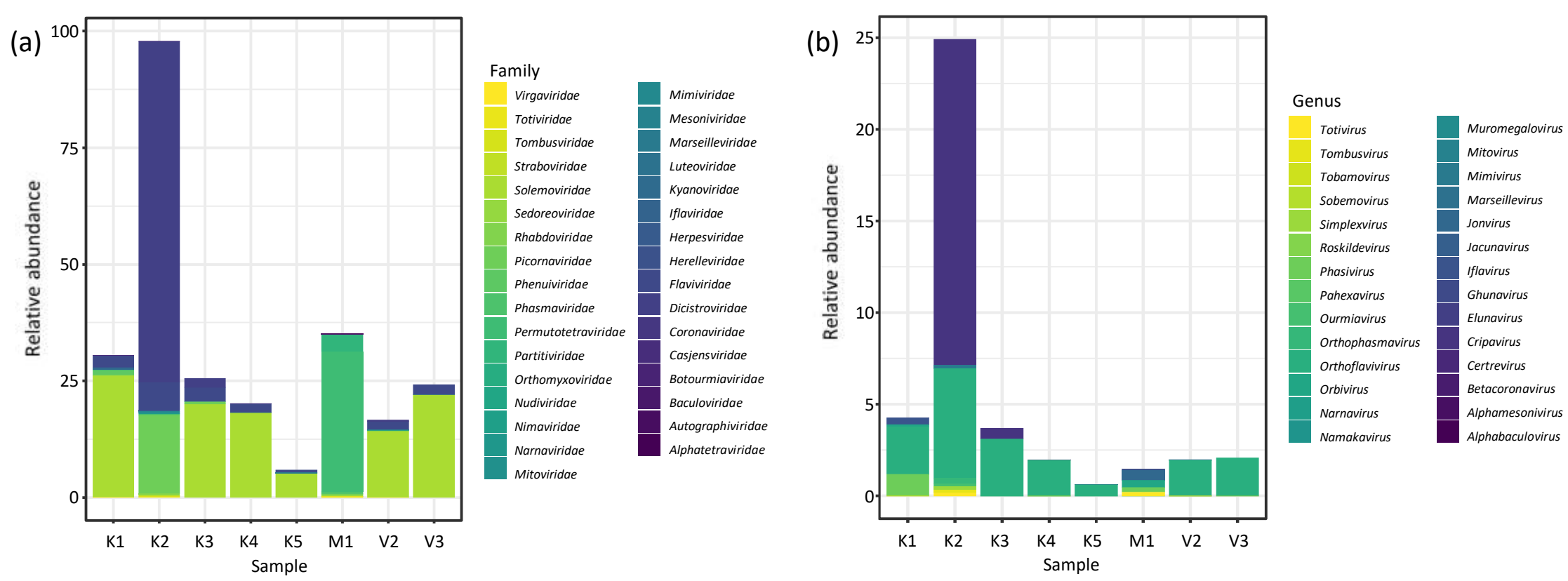

Supplementary Fig. 2 Viral families and genera detected in field mosquito pools (after filtering out those with <10 reads per million viral reads or evidence of index-hopping). a) Families in each field mosquito pool. b) Genera in each field mosquito pool. Per International Committee on Taxonomy of Viruses recommendations, relative abundance of *Orthoflavivirus* comprises both *Orthoflavivirus* and *Flavivirus* identified by KrakenUniq.

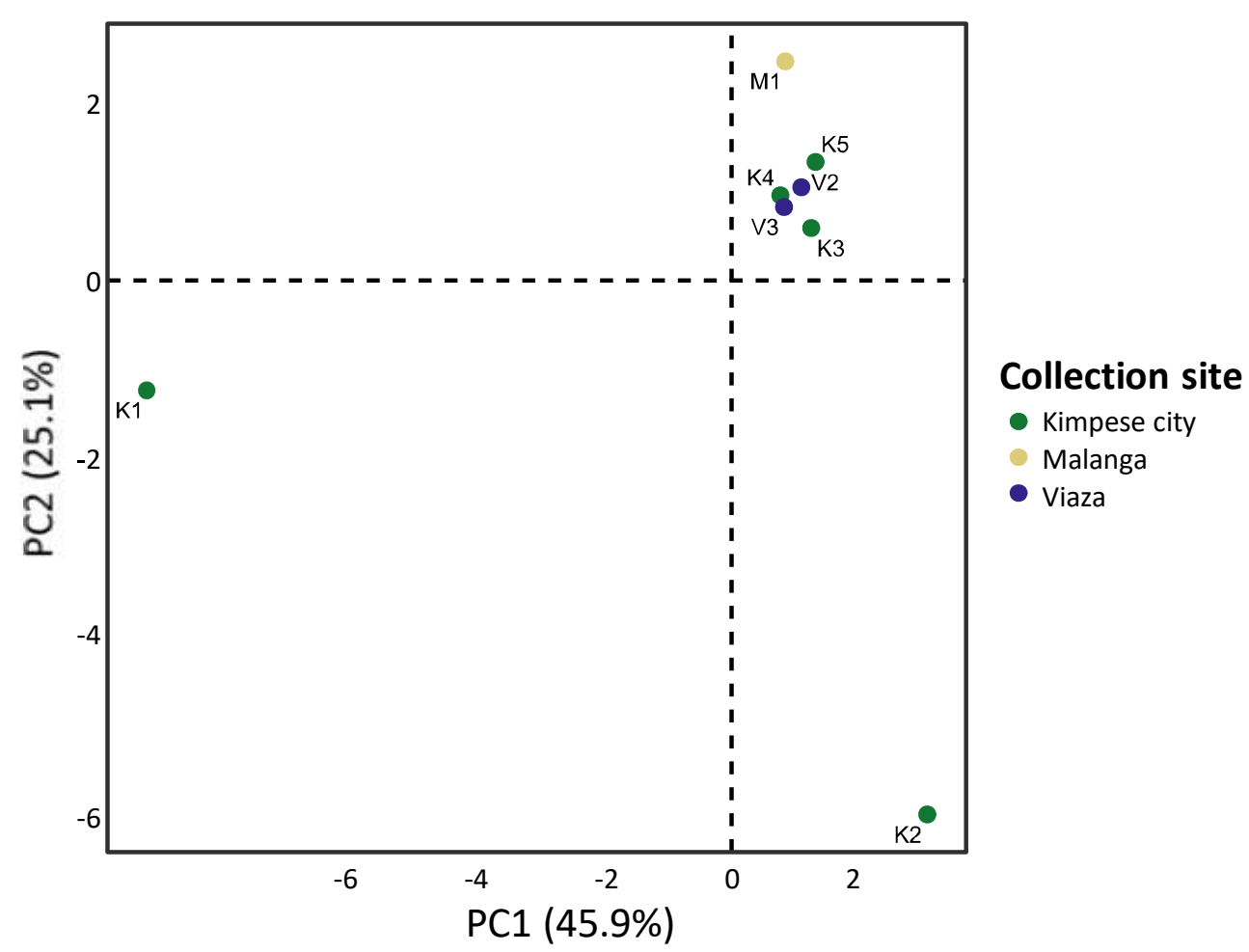

Supplementary Fig. 3 Principal component analysis (PCA) annotated by collection site using a data matrix containing relative abundance of viral genera (after applying the 10 reads per million viral reads threshold and index-hopping filter).

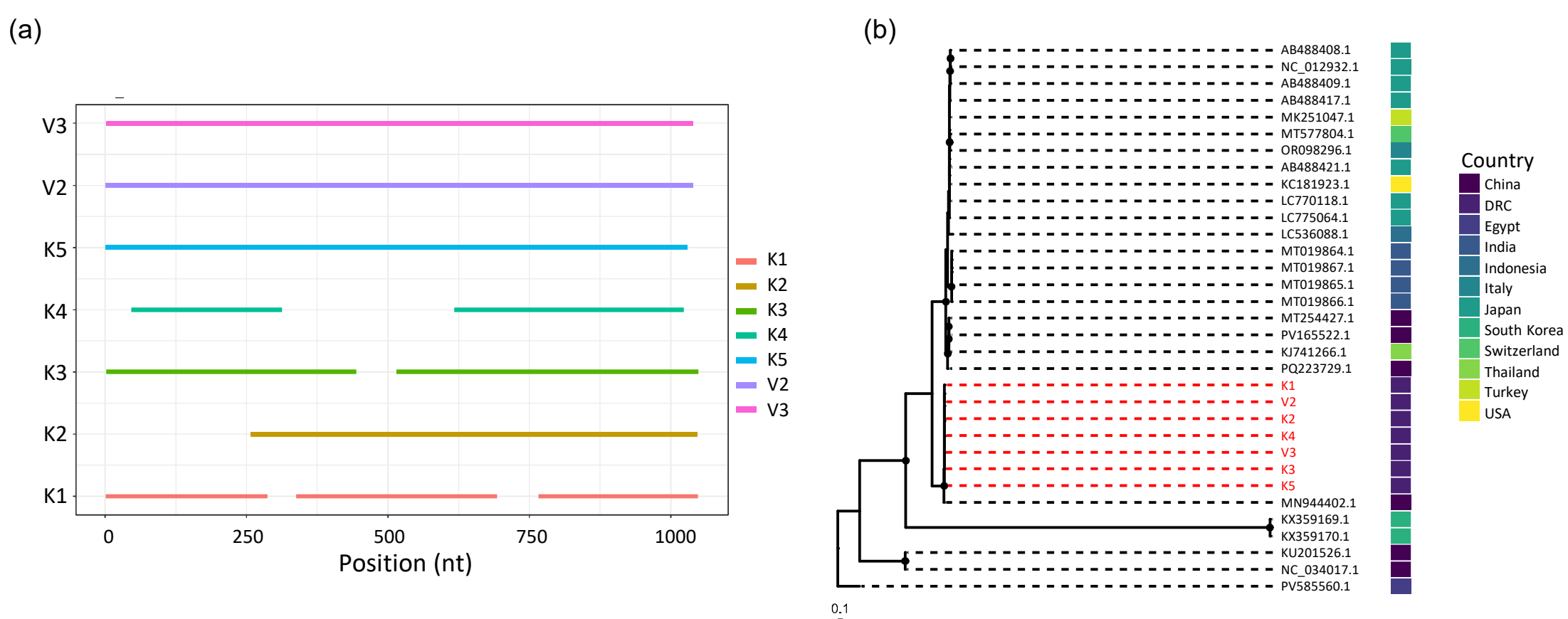

Supplementary Fig. 4 Coverage plot and phylogenetic analysis of *Aedes flavivirus* reads in DRC field mosquito pools. a) Coverage of *Aedes flavivirus* consensus sequences from 7 mosquito pools (x-axis: genomic coordinates of the published *Aedes flavivirus* sequence MN944402.1), b) *Aedes flavivirus* consensus sequences (red) compared to published sequences (black), constructed using RAxML-NG with the TIM2+F+G4 model and PV585560.1 as outgroup. Analysis of sequences from pools mapping to the polyprotein region (MN944402.1, 1-1049, 1049bp). Nodes with >80% bootstrap support across 1,000 replicates are annotated by a black circle.

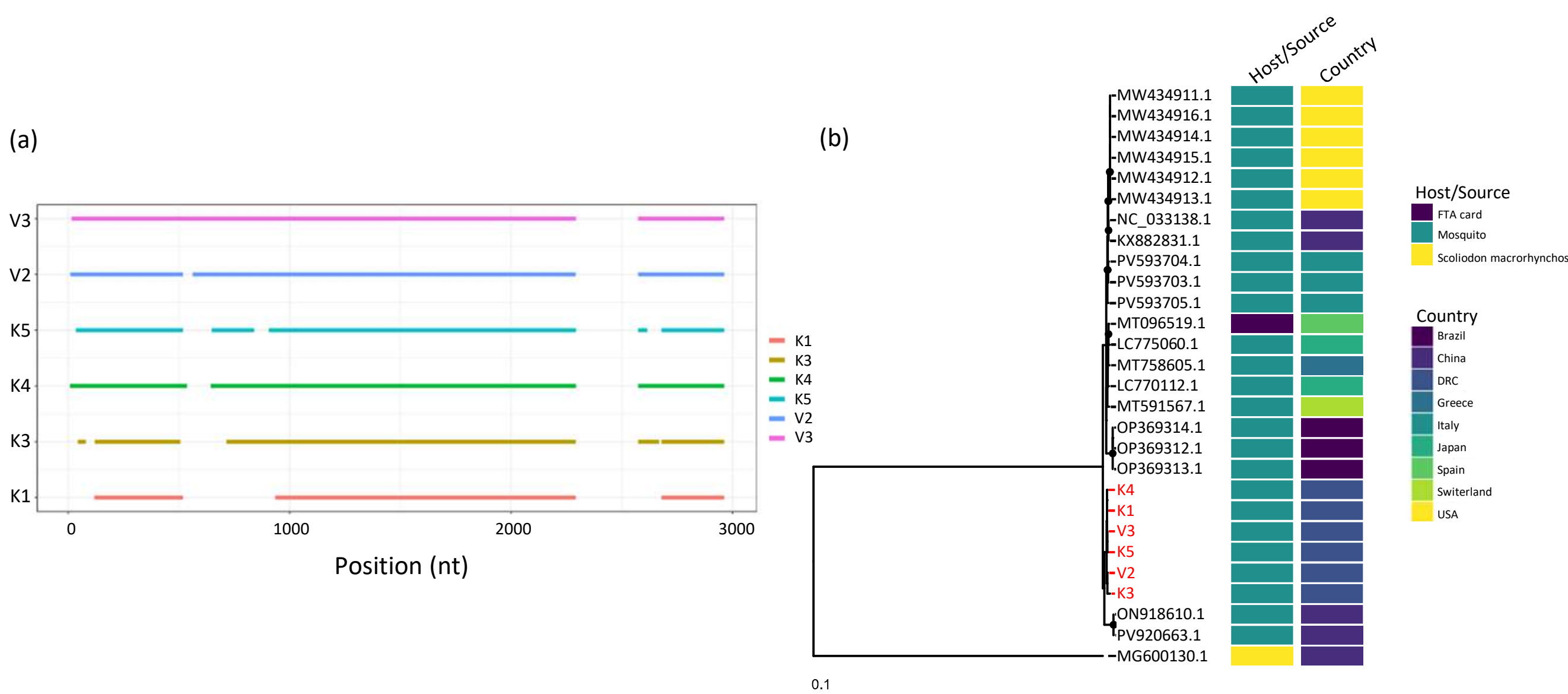

Supplementary Fig. 5 Coverage plot and phylogenetic analysis of *Wenzhou sobemo-like virus 4* reads in DRC field mosquito pools. a) Coverage of *Wenzhou sobemo-like virus 4* consensus sequences from 6 mosquito pools (x-axis: genomic coordinates of the published sequence ON918610.1), b) *Wenzhou sobemo-like virus 4* consensus sequences (red) compared to published sequences (black), constructed using RAxML-NG with the SYM+R3 model and MG600130.1 as outgroup. Analysis of sequences from pools including the hypothetical protein 1 (ON918610.1, 69-1838 nt) and hypothetical protein 2 (ON918610.1, 1580-2905 nt) regions. Nodes with >80% bootstrap support across 1,000 replicates are annotated by a black circle.

(a)

(b)

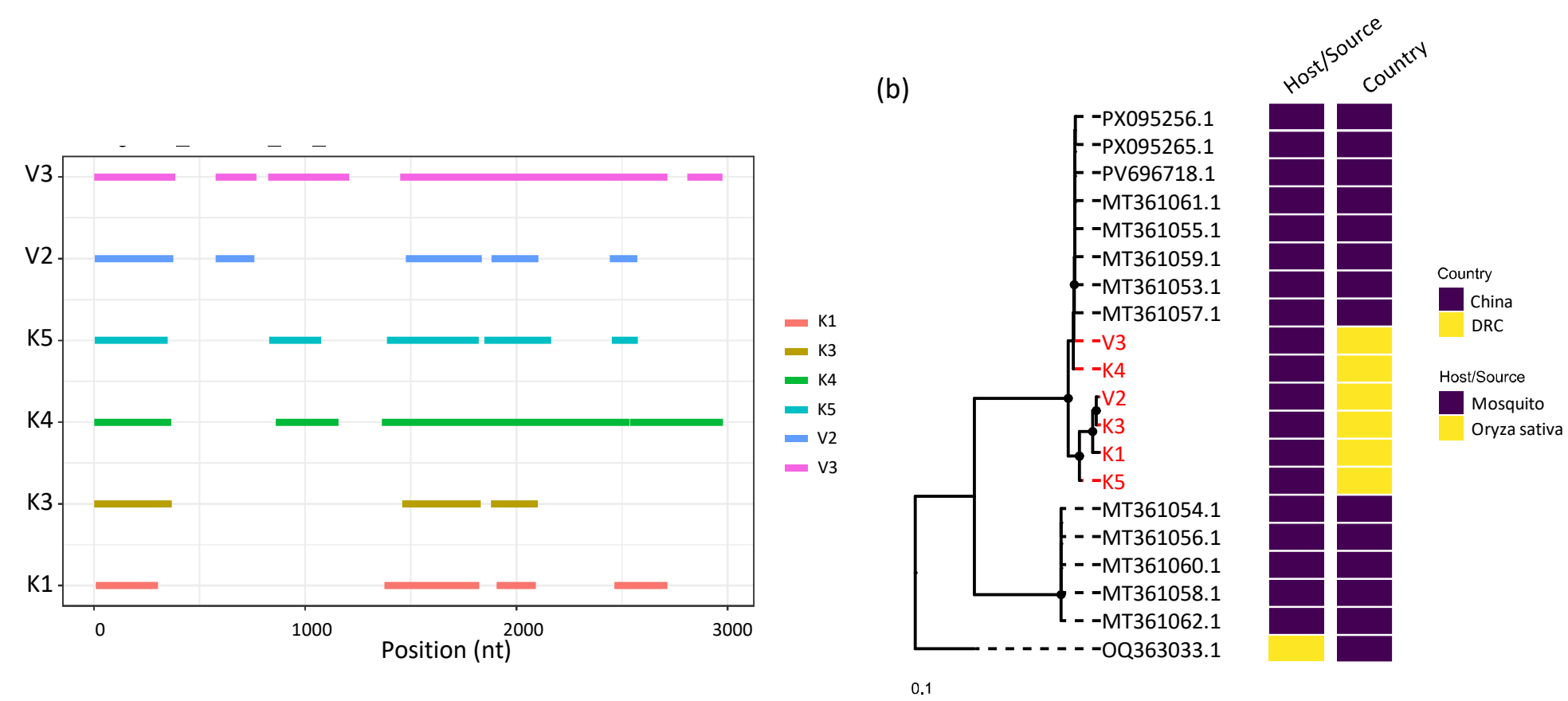

Supplementary Fig. 6 Coverage plot and phylogenetic analysis of *Guangzhou sobemo-like virus* reads in DRC field mosquito pools. a) Coverage of *Guangzhou sobemo-like virus* consensus sequences from 6 mosquito pools (x-axis: genomic coordinates of the published sequence MT361055.1), b) *Guangzhou sobemo-like virus* consensus sequences (red) compared to published sequences (black), constructed using RAxML-NG with the HKY+F model and OQ363033.1 as outgroup. Analysis of sequences from pools including the *orf1* (MT361055.1, 71-1840 nt) and *orf2* gene (MT361055.1, 1582-2907 nt) regions. Nodes with >80% bootstrap support across 1,000 replicates are annotated by a black circle.

(a)

(b)

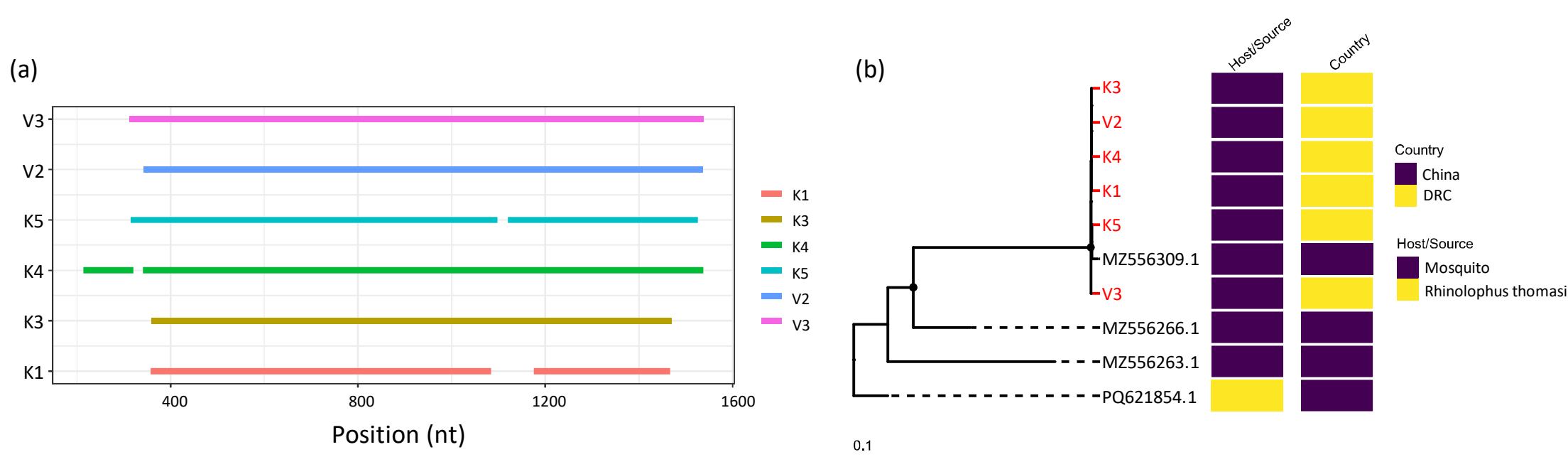

Supplementary Fig. 7 Coverage plot and phylogenetic analysis of *Sichuan mosquito sobemo-like virus* reads in DRC field mosquito pools. a) Coverage of *Sichuan mosquito sobemo-like virus* consensus sequences from 6 mosquito pools (x-axis: genomic coordinates of the published sequence MZ556309.1), b) *Sichuan mosquito sobemo-like virus* consensus sequences (red) compared to published sequences (black), constructed using RAxML-NG with the TPM2+F+I model and PQ621854.1 as outgroup. Analysis of sequences from pools including the capsid protein (MZ556309.1, 214-720 nt) and hypothetical protein (MZ556309.1, 823-1538 nt) regions. Nodes with >80% bootstrap support across 1,000 replicates are annotated by a black circle.

(a)

(b)

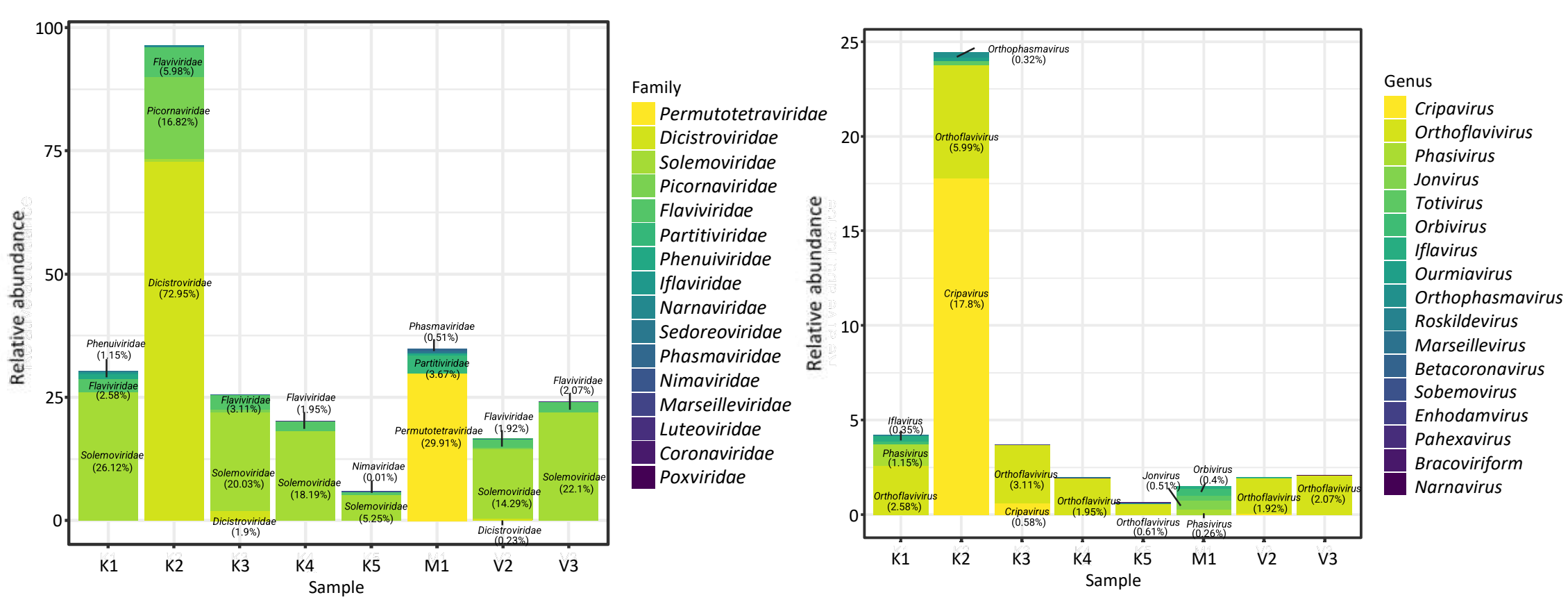

Supplementary Fig. 8 Viral families and genera in field mosquito pools (before filtering out those with <10 reads per million viral reads or evidence of index-hopping). a) Top five families in each field mosquito pool; the top three families with relative abundance >0.1% in each pool are labeled. b) Top five genera in field mosquito pools; the top three genus with relative abundance >0.1% in each pool are labeled. Per International Committee on Taxonomy of Viruses recommendations, relative abundance of *Orthoflavivirus* comprises both *Orthoflavivirus* and *Flavivirus* identified by KrakenUniq.

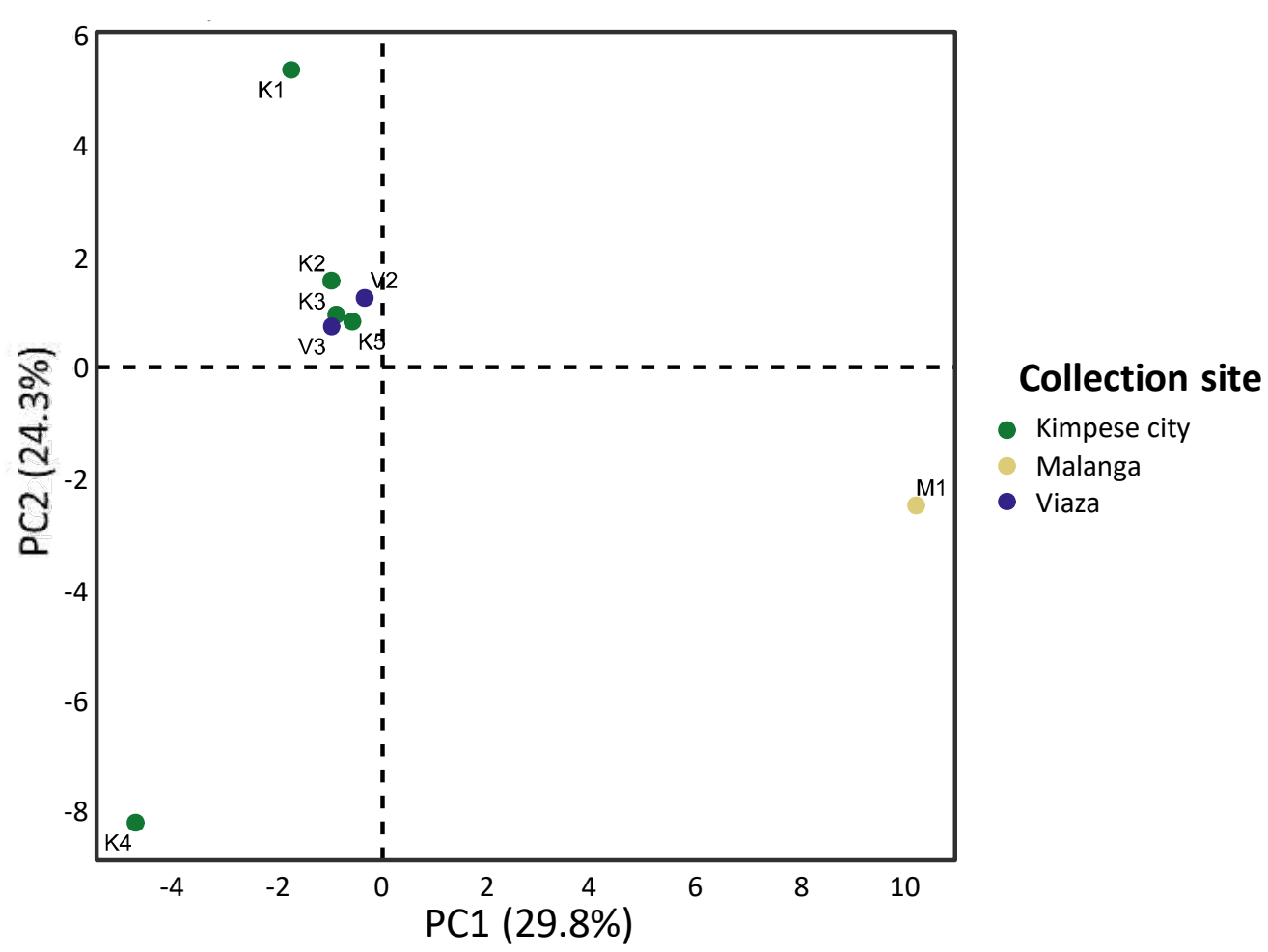

Supplementary Fig. 9 Principal component analysis (PCA) annotated by collection site using a data matrix containing relative abundance of viral genera (before applying the 10 reads per million viral reads threshold and index-hopping filter).

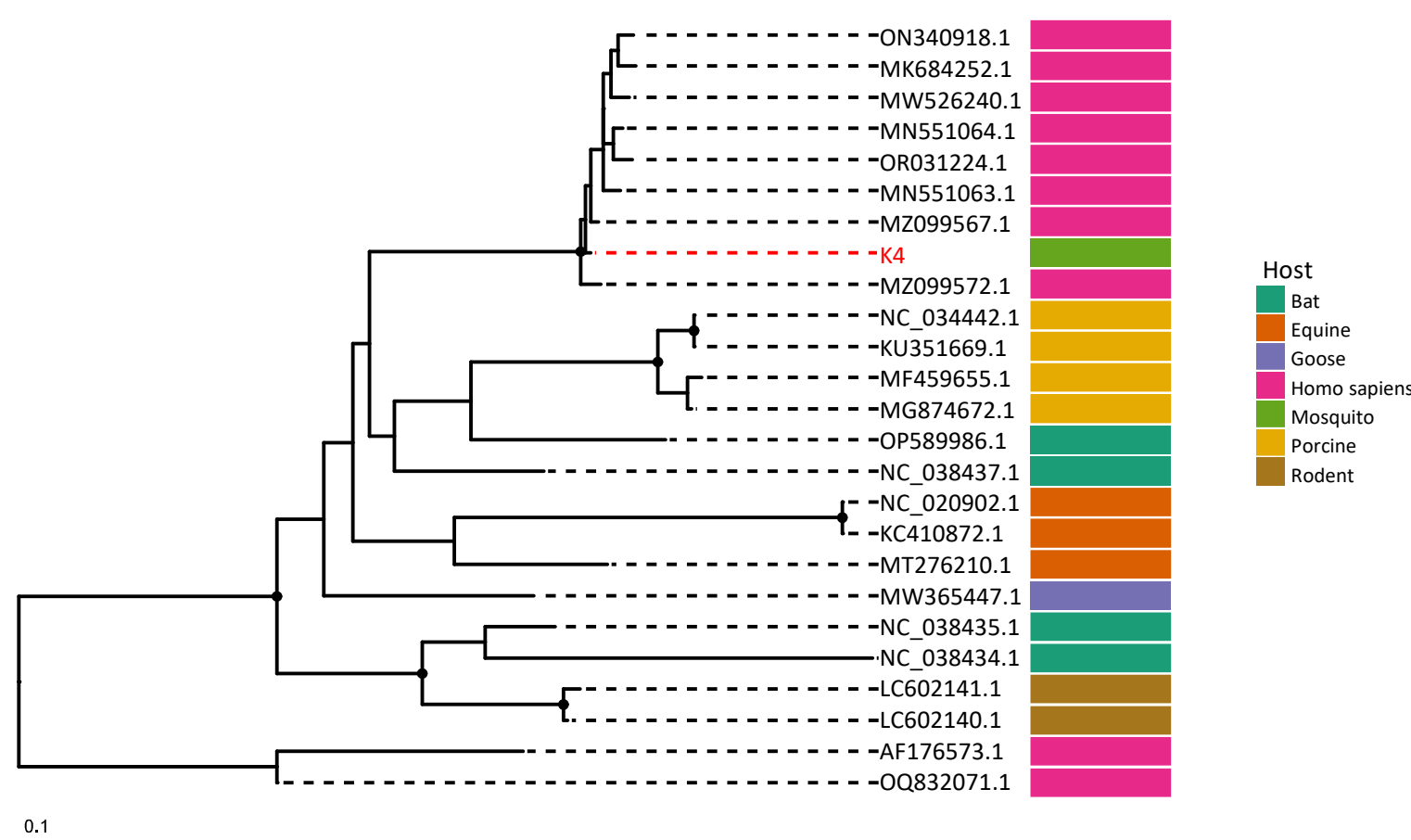

Supplementary Fig. 10 Phylogenetic analysis of pegivirus sequence from a DRC field mosquito pool (red) compared to the polyprotein gene region of published sequences (black), constructed using RAxML-NG with the HKY+F+G4 model and hepatitis C virus sequences (AF176573.1 and OQ832071.1) as outgroups. Nodes with >80% bootstrap support across 1,000 replicates are annotated by a black circle.

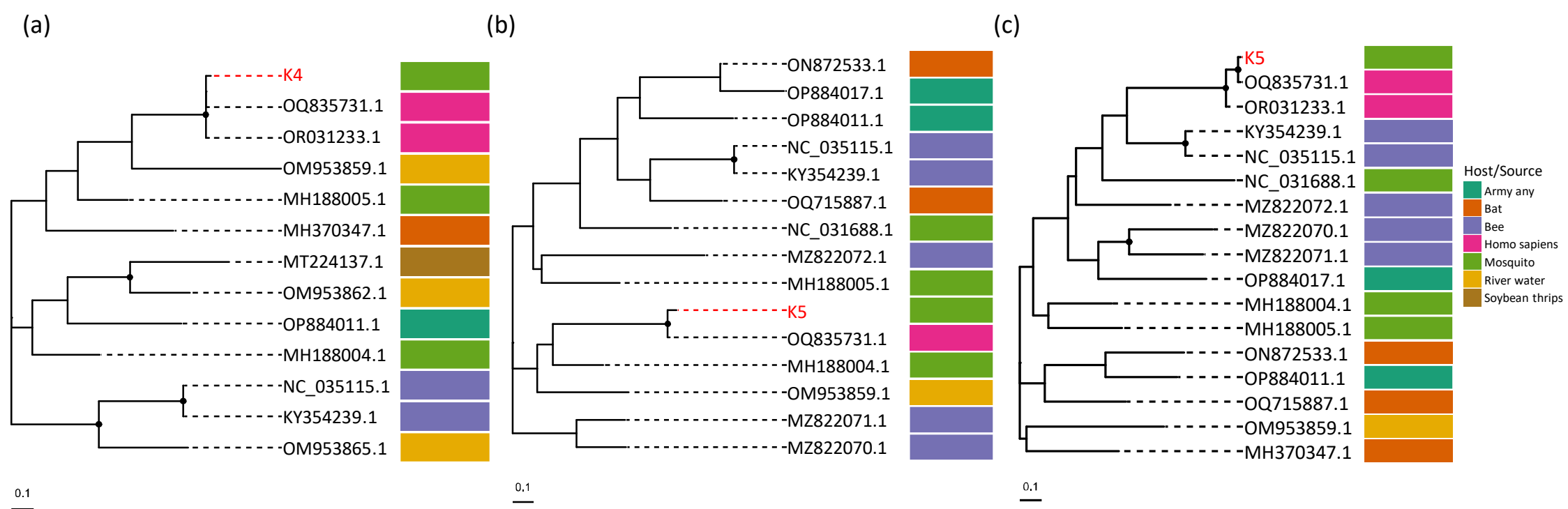

Supplementary Fig. 11 Phylogenetic analysis of *human blood-associated dicistrovirus* sequences in DRC field mosquito pools (red) compared to published sequences (black), constructed using RAxML-NG with the HKY+F+I, HKY+F, and TPM3+F+R2 models, respectively. Analysis of reads from pools a) K4 and b) K5 mapping to the open reading frame 2 (ORF2) region, and c) from K5 mapping to the open reading frame 1 (ORF1) region. Nodes with >80% bootstrap support across 1,000 replicates are annotated by a black circle.

SUPPLEMENTARY TABLES

Supplementary Table 1. Mosquito species identified by morphology at each collection site

Location	*Aedes albopictus*	*Aedes aegypti*	*Aedes simpsoni*	Total
Kimpese city	239	1	2	242
Malanga	312	3	1	316
Viaza	102	4	0	106
Total	653	8	3	664

Supplementary Table 2. Mosquito species identification using Nanopore sequencing data

Sample name	Absolute number of classified reads mapping to *Aedes albopictus*	Absolute number of classified reads mapping to *Aedes aegypti*	Absolute number of classified reads mapping to *Aedes simpsoni*
K1	114	3	0
K2	412	8	0
K3	167	5	0
K4	145	5	0
K5	172	5	0
M1	43	6	0
V2	151	3	0
V3	175	1	0

Supplementary Table 3. Microbial genera in the laboratory uninfected *Ae. aegypti* mosquito pool and water control identified using the krakenUniq default nt database (de novo assembled contigs, generated from raw reads after human and mosquito filtering)

Laboratory mosquito pool	Water control
Chryseobacterium	*Acinetobacter*
Pseudomonas	*Cutibacterium*
Enterobacter	*Herbaspirillum*
Penicillium	*Methylophilus*
Cupriavidus	*Corynebacterium*
Aspergillus	*Gemella*
Azospirillum	*Hathewaya*
Staphylococcus	*Malassezia*
Leucobacter	*Ralstonia*
Wallemia	*Delftia*
Acinetobacter	*Inhella*
Variovorax	*Photorhabdus*
Comamonas	*Sphingomonas*
Sphingomonas	*Nocardioides*
Dyadobacter	*Mycobacterium*
Marinilongibacter	*Mycolicibacillus*
Siphonobacter	*Kocuria*
Pedobacter	*Rothia*
Gordonia	*Modestobacter*
Rhodococcus	*Kutzneria*
Betanodavirus	*Staphylococcus*
	Streptococcus
	Veillonella
	Peptoniphilus
	Cloacibacterium
	Babesia
	Natronomonas

Supplementary Table 4. Viral genera identified in laboratory uninfected *Ae. aegypti* mosquito pool and water control using KrakenUniq with default nt database (reads could not be *de novo* assembled to contigs)

Laboratory mosquito pool	Water control
Quaranjavirus	*Pahexavirus*
Betanodavirus	*Elvirus*
	Sextaecvirus
	Andhravirus
	Schiekvirus
	Samunavirus
	Haloferacalesvirus
	Vojvodinavirus
	Sanovirus
	Gammaretrovirus
	Picobirnavirus
	Betacoronavirus
	Allexivirus
	Parapoxvirus
	Mimivirus
	Inovirus
	Betapapillomavirus

Supplementary Table 5. Top ten viral genera contributing to principal components 1 (PC1) and 2 (PC2) during principal component analysis (PCA)

PC1	PC2
Certrevirus	*Orthophasmavirus*
Elunavirus	*Orthoflavivirus*
Ghunavirus	*Cripavirus*
Jacunavirus	*Mimivirus*
Mitovirus	*Simplexvirus*
Namakavirus	*Tombusvirus*
Narnavirus	*Ourmiavirus*
Roskildevirus	*Alphamesonivirus*
Phasivirus	*Jonvirus*
Iflavirus	*Alphabaculovirus*

Supplementary Table 6. DENV read counts, reads per million viral reads identified using the KrakenUniq nt database, and multiplex real-time PCR results using the pan-DENV assay published by Waggoner *et al.* (*Emerg Infect Dis*. 2016)

Sample	DENV reads, n	DENV reads/million viral reads	Coordinates [reference]	DENV real-time PCR
K1	1	10.3	1-90[1]	Negative
K2	0	0	0	Negative
K3	14	31.9	1-91[1]	**Positive**
K4	83	68.7	1-93[1]	**Positive**
K5	65	27.7	1-92[1], 10667-10761[2]	Negative
M1	0	0	0	Negative
V2	28	17.3	1-91[1]	Negative
V3	114	32.1	1-97[1]	**Positive**

[1] Based on published DENV-4 sequence: MG601754.1
[2] Based on published DENV-2 sequence: MH048672.1

Supplementary Table 7. Read counts of *bat faecal associated dicistrovirus 4*

Sample	*Bat faecal associated dicistrovirus 4* reads, n	Coordinates[1]	Total viral reads (based on nt database)	*Bat faecal associated dicistrovirus 4* reads/million viral reads, n (based on nt database)
K1	0	0	96956	0
K2	33	7078-7330	2472	13349.5
K3	182	7078-7330	439000	414.6
K4	0	0	1207424	0
K5	0	0	2204216	0
M1	1	0	1316904	0
V2	52	7078-7330	1618920	32.1
V3	0	0	3555364	0

[1] Based on published Bat faecal associated dicistrovirus 4 sequence: ON872534.1

Supplementary Table 8. Read counts of the most prevalent insect-specific viruses (reads/million reads mapping to viruses)

	K1	K2	K3	K4	K5	M1	V2	V3
Aedes flavivirus	1,320	58,252	656	268	230	0	284	71
Wenzhou sobemo-like virus 4	246,875	0	314,077	356,127	53,343	0	211,721	320,021
Hubei mosquito virus 2	330	0	228	278	76	604,697	337,739	9,509
Guangzhou sobemo-like virus	160,691	1,618	148,055	96,652	30,291	0	103,348	145,950
Sichuan mosquito sobemo-like virus	98,890	1,618	51,417	83,487	21,927	0	39,073	74,002

Supplementary Table 9. *Aedes flavivirus* consensus sequence coverage and nucleotide identity by sample using BLAST

Sample	Reference (Accession, country)	Aligned length (bp)	Reference length (bp)	Coverage of reference (%)	Nucleotide identity (%)
K1	MN944402.1, China	927	1050	88.28	99.13
K2	MN944402.1, China	791	1050	75.33	98.86
K3	MN944402.1, China	987	1050	93.14	97.97
K4	MN944402.1, China	677	1050	64.48	99.11
K5	MN944402.1, China	1029	1050	98.00	99.03
V2	MN944402.1, China	1040	1050	99.05	99.23
V3	MN944402.1, China	1039	1050	98.95	99.04

Supplementary Table 10. *Wenzhou sobemo-like virus 4* consensus sequence coverage and nucleotide identity by sample using BLAST

Sample	Reference (Accession, country)	Aligned length (bp)	Reference length (bp)	Coverage of reference (%)	Nucleotide identity (%)
K1	ON918610.1, China	2070	2962	68.88	93.86
K3	ON918610.1, China	2408	2962	81.30	94.19
K4	ON918610.1, China	2576	2962	86.97	94.72
K5	ON918610.1, China	2402	2962	81.09	94.63
V2	ON918610.1, China	2638	2962	89.06	95.07
V3	ON918610.1, China	2675	2962	90.31	94.73

Supplementary Table 11. *Guangzhou sobemo-like virus* consensus sequence coverage and nucleotide identity by sample using BLAST

Sample	Reference (Accession, country)	Aligned length (bp)	Reference length (bp)	Coverage of reference (%)	Nucleotide identity (%)
K1	MT361055.1, China	1194	3107	38.43	98.83
K3	MT361055.1, China	965	3107	31.06	98.86
K4	MT361055.1, China	2277	3107	73.29	99.21
K5	MT361055.1, China	1492	3107	48.02	97.65
V2	MT361055.1, China	1277	3107	41.10	98.98
V3	MT361055.1, China	2402	3107	77.31	98.88

Supplementary Table 12. *Sichuan mosquito sobemo-like virus* consensus sequence coverage and nucleotide identity by sample using BLAST

Sample	Reference (Accession, country)	Aligned length (bp)	Reference length (bp)	Coverage of reference (%)	Nucleotide identity (%)
K1	MZ556309.1, China	1020	1627	62.69	96.08
K3	MZ556309.1, China	1113	1627	64.41	95.88
K4	MZ556309.1, China	1325	1627	81.44	94.88
K5	MZ556309.1, China	1212	1627	74.49	94.55
V2	MZ556309.1, China	1196	1627	73.51	95.90
V3	MZ556309.1, China	1227	1627	75.41	95.68

Supplementary Table 13. Primers for mosquito species determination and blood meal investigation. Reaction mixtures and reaction conditions are the same for all assays.

<table>
<tr><th>Target</th><th>Primer sequences 5′ to 3′</th><th>Study</th><th>Reaction mixture</th><th>Reaction Condition</th></tr>
<tr><td>16S</td><td>CGGTTGGGGTGACCTCGGA
GCTGTTATCCCTAGGGTAACT</td><td>Taylor, 1996</td><td rowspan="5">5 µl 5× PrimeSTAR GXL Buffer (Takara Bio)
1 µl PrimeSTAR GXL DNA Polymerase (Takara Bio)
4 µl dNTP Mixture (2.5 mM each)
200 nM Primer 1
200 nM Primer 2
2 µl Template
Add H_2O to 50 µl</td><td rowspan="5">35 cycles of:
98°C for 10 s
55°C for 15 s
68°C for 1 min;
Final extension at 68°C for 2 min;
Hold at 4°C</td></tr>
<tr><td>cytB</td><td>CCATCCAACATCTCAGCATGATGAAA
GCCCCTCAGAATGATATTTGTCCTCA</td><td>Meyer, Hofelein, Luthy, & Candrian, 1995</td></tr>
<tr><td>COX1</td><td>TACAGTTGGAATAGACGTTGATAC
TCCAATGCACTAATCTGCCATATTA</td><td>Zhang & Hewitt, 1996</td></tr>
<tr><td>COI</td><td>GGTCAACAAATCATAAAGATATTGG
TAAACTTCAGGGTGACCAAAAAATCA</td><td>Folmer, 1994</td></tr>
<tr><td>ITS2</td><td>GCTCGTGGATCGATGAAGAC
TGCTTAAATTTAGGGGGTGTAGTCAC</td><td>Batovska, Blacket, Brown, & Lynch, 2016</td></tr>
</table>

Supplementary Table 14. Published probes by Fauver *et al*. (*Virology.* 2018) for mosquito ribosomal RNA depletion

Primer Name	Sequence 5′ to 3′	Primer Name	Sequence 5’ to 3’
12s mtDNA (223-249) Rev.	TTGTGCCAGCTACCGCGGTTATACAAA	LSU (3875-3901) Rev.	GGATCAAAAAGCCACGTCGCTATGAAC
12s mtDNA (552-580) Rev.	ATATTCTGATGACGGCGATATACAAATT	LSU (3992-4018) Rev.	GGGTAAAACTAACCTGTCTCACGACGG
12s mtDNA (621-644) Rev.	ATAAACTGCACCTTGACCTGACAT	LSU (402-429) Rev.	TGCTGCACTATCAAGCAACACGACTCCA
12s mtDNA (820-850) Rev.	TCATTCTAGATACACTTTCCAGTACATCTAC	LSU (4070-4095) Rev.	GGTGTCGAAACTAGGTATATCCACGT
16s mtDNA (1161-1136) Rev.	TCTTAATCCAACATCGAGGTCGCAAT	LSU (4148-4175) Rev.	GACCTTAGAGGCGTTCAGGCATAATCCA
16s mtDNA (1219-1245) Rev.	GCTTACGCCGGTTTGAACTCAGATCAT	LSU (4209-4230) Rev.	CGGTCGGACAAGCGGGTAACAC
16s mtDNA (170-199) Rev.	CCTGATACACAAGGTACAATAAATTAAATT	LSU (4300-4273) Rev.	CTACACTCAACTAAGTACACTCTTGCTC
16s mtDNA (721-745) Rev.	TCAGTGGGCAGGTTAGACTTTAAAT	LSU (4321-4347) Rev.	GCGTTCCAGTATCTTAGATACTTGCTG
16s mtDNA (907-935) Rev.	AAATAAAGATCTATAGGGTCTTCTCGTC	LSU (4450-4476) Rev.	AAGCAAGGGTAGTCTGAGAGGATCGAA
LSU (1210-1235) Rev.	AGGCATAGTTCACCATCTTTCGGGTC	LSU (4495-4522) Rev.	CACTAGTGTTACGTGTTCCGCTCCATAC
LSU (1319-1345) Rev.	ACTAGATGGTTCGATTGGTCTTTCGCC	LSU (4966-4989) Rev.	CGCTAGCTTCCGAACACCTAATGG
LSU (1538-1562) Rev.	GAGTGCTTTTCAACTTTCCCTCACG	LSU (505-533) Rev.	TTGACTCTCTATTCAGAGTGCTTTTCAAC
LSU (1610-1636) Rev.	CACAGCACCAGTTCTGCTTACCAAAAC	LSU (5188-5210) Rev.	TCTACAACTCGCAGCTCCCAGCC
LSU (1707-1727) Rev.	TAGCACGAGGCCAACCGCTGT	LSU (999-971) Rev.	CGCAAGATAGACTTCTTGGTCCGTGTTTC
LSU (1743-1771) Rev.	GTGAGTTGTTACACACTCCTTAGCGGATG	SSU (1047-1070) Rev.	TCTACGAATTTCACCTCTCGCGCC
LSU (1769-1794) Rev.	GAGCAGCTACCCTACCCTTACAGAGG	SSU (1224-1247) Rev.	GGTTTCCCGGAAGCTACTGAGAGC
LSU (1779-1804) Rev.	GGTCAGTGTAGAGCAGCTACCCTACC	SSU (1292-1314) Rev.	TGGTGCCCTTCCGTCAATTCCTT
LSU (1844-1864) Rev.	CAGCACACCAGAGCTCGGTCG	SSU (1330-1347) Rev.	CGTACACGGACGGTCCCA
LSU (1925-1945) Rev.	CATCGCCGAACGAAGGATTCG	SSU (1413-1435) Rev.	CGGCCATGCACCACTACCCTTAA
LSU (1938-1966) Rev.	CCAAGATCTGTGCCAGTGGCGGCTCCATG	SSU (1531-1562) Rev.	AGAACGCCCAGGTACACCATTGTGAG
LSU (196-223) Rev.	GCTCTTCCCGTTTCGCTCGCAGCTACTC	SSU (1540-1565) Rev.	GAACGCCCAGGTACACCATTGT
LSU (2272-2300) Rev.	CTCGAAGAAAAGGATTCATGTTGCCATGA	SSU (1606-1627) Rev.	CGAACAAGACCTTGACCGACGA
LSU (2492-2520) Rev.	GCTGCGGATTCGGTACAATCTGTTGAGAG	SSU (1622-1643) Rev.	CACCATGAAGCTGGGTCGAACA
LSU (2590-2615) Rev.	GCACCAGTTCTGCTTACCAAAACTTG	SSU (1696-1721) Rev.	AGCCCAGAACATCTAAGGGCATCACG
LSU (2601-2628) Rev.	GCCAATCCTTGTCCCGAAGTTACGGATC	SSU (1750-1776) Rev.	GTGTGTACAAAGGGCAGGGACGTAATC
LSU (2788-2813) Rev.	GCAATGTATAGGCAAACGACTTGAGC	SSU (1923-1955) Rev.	TTGGCAAACGCTTTCGCTTCTG
LSU (2820-2845) Rev.	CCCTCAGCCGTGCCAGTTCTGAATTG	SSU (2001-2028) Rev.	CTCTAAATCATCAAGTTCGGTCAACTTC

LSU (2925-2949) Rev.	CTACCACCAAGATCTGTGCCAGTGG	SSU (2046-2069) Rev.	CGCAGGTTCACCTACGGAAACCTT
LSU (2939-2965) Rev.	TTTACCCGCGCTTGCTTGAATTTCTTC	SSU (2054-2080) Rev.	CGATTTTGGTTTCCCGGAAGC
LSU (3019-3045) Rev.	TCGTTAATCCATTCATGCGCGTCACTA	SSU (2177-2198) Rev.	TTGTGGTGCCCTTCCGTCAATT
LSU (3041-3063) Rev.	GGGTTACGACTGGATTCCCATGC	SSU (2522-2545) Rev.	GCATCACGGACCTGTTATCGCT
LSU (3057-3079) Rev.	CCTTTCGCCGACTGATGGGTTAC	SSU (3218-3235) Rev.	CCAGTGCGTCGGATGACC
LSU (3120-3140) Rev.	CACGACGATGCTACGATGCAG	SSU (324-350) Rev.	ACTACCATCAATAGTTGATAGGGCAGA
LSU (3223-3250) Rev.	AGGCAACAGTAAGAGTGGTGGTATCTCA	SSU (3459-3481) Rev.	GCACTCAAGAATGTGTGCATCGG
LSU (3240-3264) Rev.	GGCAAACGTGACATCATAAACGATC	SSU (408-431) Rev.	GTGGTAGCCATTTCTCAGGCTCCC
LSU (3359-3386) Rev.	CGACATGACTGAACGCTGAACAAGAAAC	SSU (562-585) Rev.	CCAGACTTGCCCTCCACTTGATCC
LSU (3380-3401) Rev.	GAGCTGACCTTTGGACACCTCC	SSU (80-107) Rev.	GCTGTTATACTGAGCCTTATGCGGTTTC
LSU (3541-3567) Rev.	CACAAGCCAGTTATCCCTGTGG	SSU (841-863) Rev.	CCAACAGTCCCGGCATAATGC
LSU (3633-3659) Rev.	GTACCGCCCCAGTCAAACTCCGCACCT	SSU (859-882) Rev.	AGCACTCTAATTTGTTCAAGGTAA
LSU (3722-3748) Rev.	TCAAGCCAGCTTTTGTCCTTATGCTCA	SSU (979-1006) Rev.	CGCAAGCGACCGTACGATCG

Supplementary Table 15. Published primers and probes for DENV real-time PCR

Name	Primer sequences 5′ to 3′	Reaction mixture	Reaction Conditions	Study
1, -2, -3 Forward	CAGATCTCTGATGAACAACCAACG	2 µl SuperScript™ III RT/Platinum™ Taq Mix (Life Technologies)<br>25 µl 2X Reaction Mix (Life Technologies)<br>5 µl Template<br>350 nM 1, -2, -3 Forward primer<br>350 nM 2 Forward C to T primer<br>300 nM 3 Forward C to T primer<br>450 nM 4 Forward primer<br>300 nM 1, 3 Reverse primer<br>350 nM 2 Reverse primer<br>350 nM 2 Reverse A to G primer<br>450 nM 4 Reverse primer<br>100 nM Probe A<br>100 nM Probe B<br>100 nM Probe C<br>100 nM Probe D<br>Add H_2O to 50 µl	52°C for 15 min;<br>94°C for 2 min;<br>45 cycles of:<br>94°C for 15 s,<br>55°C for 20 s (acquisition),<br>68°C for 20 s	Waggoner, 2016
2 Forward C to T	CAGATCTCTGATGAATAACCAACG			
3 Forward C to T	CAGATTTCTGATGAACAACCAACG			
4 Forward	GATCTCTGGAAAAATGAAC			
1, 3 Reverse	TTTGAGAATCTCTTCGCCAAC			
2 Reverse	AGTTGACACGCGGTTTCTCT			
2 Reverse A to G	AGTCGACACGCGGTTTCTCT			
4 Reverse	AGAATCTCTTCACCAACC			
Probe A	FAM- CTCGCGCGTTTCAGCATAT - BHQ1			
Probe B	FAM- CTCTCGCGTTTCAGCATAT - BHQ1			
Probe C	FAM- CTCTCACGTTTCAGCATATTG - BHQ1			
Probe D	FAM- CTCACGCGTTTCAGCATAT - BHQ1			

Supplementary Table 16. Sequences for *Bat faecal associated dicistrovirus 4* phylogenetic analysis

Accession number/ Sample name	Virus name	Host/Source	This study
K2	Bat faecal associated dicistrovirus 4	Mosquito	yes
K3	Bat faecal associated dicistrovirus 4	Mosquito	yes
MH188004.1	Culex dicistrovirus 1	Mosquito	no
MH188005.1	Culex dicistrovirus 2	Mosquito	no
MT195550.1	Soybean thrips dicistrovirus 1	Soybean thrips	no
MZ822070.1	Apis dicistrovirus 2	Bee	no
MZ822071.1	Apis dicistrovirus 3	Bee	no
OM953862.1	Flumine dicistrovirus 2	River water	no
OM953865.1	Flumine dicistrovirus 3	River water	no
ON872534.1	Bat faecal associated dicistrovirus 4	Bat	no
OQ715884.1	Wenzhou bat dicistrovirus 3	Bat	no
OQ715887.1	Wenzhou bat dicistrovirus 6	Bat	no
V2	Bat faecal associated dicistrovirus 4	Mosquito	yes
MH370347.1	Bat dicistrovirus	Bat	no
OP884017.1	Army ant associated dicistrovirus 7	Army ant	no
OP884013.1	Army ant associated dicistrovirus 4	Army ant	no
MT224137.1	Soybean thrips dicistrovirus 2	Soybean thrips	no
NC_029052.1	Goose dicistrovirus	Goose	no

Supplementary Table 17. Sequences for *Aedes flavivirus* phylogenetic analysis

Accession number/ Sample name	Virus name	Country	This study	Host/Source
PV585560.1	Culex flavivirus	Egypt	no	mosquito
PQ223729.1	Aedes flavivirus	China	no	mosquito
OR098296.1	Aedes flavivirus	Italy	no	mosquito
KC181923.1	Aedes flavivirus	USA	no	mosquito
PV165522.1	Aedes flavivirus	China	no	mosquito
LC775064.1	Aedes flavivirus	Japan	no	mosquito
LC770118.1	Aedes flavivirus	Japan	no	mosquito
MT577804.1	Aedes flavivirus	Switzerland	no	mosquito
MT254427.1	Aedes flavivirus	China	no	mosquito
MT019867.1	Aedes flavivirus	India	no	mosquito
MT019866.1	Aedes flavivirus	India	no	mosquito
MT019865.1	Aedes flavivirus	India	no	mosquito
MT019864.1	Aedes flavivirus	India	no	mosquito
LC536088.1	Aedes flavivirus	Indonesia	no	mosquito
NC_012932.1	Aedes flavivirus	Japan	no	mosquito
MK251047.1	Aedes flavivirus	Turkey	no	mosquito
KX359170.1	Aedes flavivirus	South Korea	no	mosquito
KX359169.1	Aedes flavivirus	South Korea	no	mosquito
KJ741266.1	Aedes flavivirus	Thailand	no	mosquito
AB488421.1	Aedes flavivirus	Japan	no	mosquito
AB488417.1	Aedes flavivirus	Japan	no	mosquito
AB488409.1	Aedes flavivirus	Japan	no	mosquito
AB488408.1	Aedes flavivirus	Japan	no	mosquito
NC_034017.1	Aedes flavivirus	China	no	mosquito
KU201526.1	Aedes flavivirus	China	no	mosquito
K1	Aedes flavivirus	DRC	yes	mosquito
K2	Aedes flavivirus	DRC	yes	mosquito
K3	Aedes flavivirus	DRC	yes	mosquito
K4	Aedes flavivirus	DRC	yes	mosquito
K5	Aedes flavivirus	DRC	yes	mosquito
V2	Aedes flavivirus	DRC	yes	mosquito
V3	Aedes flavivirus	DRC	yes	mosquito
MN944402.1	Aedes flavivirus	China	no	mosquito

Supplementary Table 18. Sequences for *Wenzhou sobemo-like virus 4* phylogenetic analysis

Accession number/ Sample name	Virus name	Country	This study	Host/Source
MG600130.1	Wenzhou tombus-like virus 1	China	no	Scoliodon macrorhynchos
OP369314.1	Wenzhou sobemo-like virus 4	Brazil	no	Mosquito
OP369313.1	Wenzhou sobemo-like virus 4	Brazil	no	Mosquito
OP369312.1	Wenzhou sobemo-like virus 4	Brazil	no	Mosquito
PV920663.1	Wenzhou sobemo-like virus 4	China	no	Mosquito
PV593705.1	Wenzhou sobemo-like virus 4	Italy	no	Mosquito
PV593704.1	Wenzhou sobemo-like virus 4	Italy	no	Mosquito
PV593703.1	Wenzhou sobemo-like virus 4	Italy	no	Mosquito
LC775060.1	Wenzhou sobemo-like virus 4	Japan	no	Mosquito
LC770112.1	Wenzhou sobemo-like virus 4	Japan	no	Mosquito
MT591567.1	Wenzhou sobemo-like virus 4	Switzerland	no	Mosquito
MT758605.1	Wenzhou sobemo-like virus 4	Greece	no	Mosquito
MW434916.1	Wenzhou sobemo-like virus 4	USA	no	Mosquito
MW434915.1	Wenzhou sobemo-like virus 4	USA	no	Mosquito
MW434914.1	Wenzhou sobemo-like virus 4	USA	no	Mosquito
MW434913.1	Wenzhou sobemo-like virus 4	USA	no	Mosquito
MW434912.1	Wenzhou sobemo-like virus 4	USA	no	Mosquito
MW434911.1	Wenzhou sobemo-like virus 4	USA	no	Mosquito
MT096519.1	Wenzhou sobemo-like virus 4	Spain	no	FTA card
NC_033138.1	Wenzhou sobemo-like virus 4	China	no	Mosquito
KX882831.1	Wenzhou sobemo-like virus 4	China	no	Mosquito
ON918610.1	Wenzhou sobemo-like virus 4	China	no	Mosquito
K1	Wenzhou sobemo-like virus 4	DRC	yes	Mosquito
K3	Wenzhou sobemo-like virus 4	DRC	yes	Mosquito
K4	Wenzhou sobemo-like virus 4	DRC	yes	Mosquito
K5	Wenzhou sobemo-like virus 4	DRC	yes	Mosquito
V2	Wenzhou sobemo-like virus 4	DRC	yes	Mosquito
V3	Wenzhou sobemo-like virus 4	DRC	yes	Mosquito

Supplementary Table 19. Sequences for *Guangzhou sobemo-like virus* phylogenetic analysis

Accession number/ Sample name	Virus name	Country	This study	Host/Source
OQ363033.1	Hangzhou sobemo-like virus 1	China	no	Oryza sativa
MT361062.1	Guangzhou sobemo-like virus	China	no	Mosquito
MT361061.1	Guangzhou sobemo-like virus	China	no	Mosquito
MT361060.1	Guangzhou sobemo-like virus	China	no	Mosquito
MT361056.1	Guangzhou sobemo-like virus	China	no	Mosquito
MT361055.1	Guangzhou sobemo-like virus	China	no	Mosquito
MT361054.1	Guangzhou sobemo-like virus	China	no	Mosquito
MT361053.1	Guangzhou sobemo-like virus	China	no	Mosquito
PX095265.1	Guangzhou sobemo-like virus	China	no	Mosquito
PX095256.1	Guangzhou sobemo-like virus	China	no	Mosquito
PV696718.1	Guangzhou sobemo-like virus	China	no	Mosquito
MT361059.1	Guangzhou sobemo-like virus	China	no	Mosquito
MT361058.1	Guangzhou sobemo-like virus	China	no	Mosquito
MT361057.1	Guangzhou sobemo-like virus	China	no	Mosquito
K1	Guangzhou sobemo-like virus	DRC	yes	Mosquito
K3	Guangzhou sobemo-like virus	DRC	yes	Mosquito
K4	Guangzhou sobemo-like virus	DRC	yes	Mosquito
K5	Guangzhou sobemo-like virus	DRC	yes	Mosquito
V2	Guangzhou sobemo-like virus	DRC	yes	Mosquito
V3	Guangzhou sobemo-like virus	DRC	yes	Mosquito

Supplementary Table 20. Sequences for *Sichuan mosquito sobemo-like virus* phylogenetic analysis

Accession number/ Sample name	Virus name	Country	This study	Host/Source
PQ621854.1	Yunnan solemo-like virus	China	no	Rhinolophus thomasi
MZ556263.1	Sichuan mosquito sobemo-like virus	China	no	Mosquito
MZ556266.1	Sichuan mosquito sobemo-like virus	China	no	Mosquito
K1	Sichuan mosquito sobemo-like virus	DRC	yes	Mosquito
MZ556309.1	Sichuan mosquito sobemo-like virus	China	no	Mosquito
K3	Sichuan mosquito sobemo-like virus	DRC	yes	Mosquito
K4	Sichuan mosquito sobemo-like virus	DRC	yes	Mosquito
K5	Sichuan mosquito sobemo-like virus	DRC	yes	Mosquito
V2	Sichuan mosquito sobemo-like virus	DRC	yes	Mosquito
V3	Sichuan mosquito sobemo-like virus	DRC	yes	Mosquito

Supplementary Table 21. Sequences for *Human pegivirus* phylogenetic analysis

Accession number/ Sample name	Virus name	Host	This study
K4	Human pegivirus	Mosquito	yes
MZ099572.1	Human pegivirus	Homo sapiens	no
ON340918.1	Pegivirus C	Homo sapiens	no
MK684252.1	Human pegivirus	Homo sapiens	no
MW526240.1	Human pegivirus	Homo sapiens	no
MZ099567.1	Human pegivirus	Homo sapiens	no
MN551063.1	Human pegivirus	Homo sapiens	no
OR031224.1	Pegivirus C	Homo sapiens	no
MN551064.1	Human pegivirus	Homo sapiens	no
NC_038437.1	Pegivirus I	Bat	no
MW365447.1	Goose pegivirus	Goose	no
KU351669.1	Pegivirus suis	Porcine	no
NC_034442.1	Pegivirus K	Porcine	no
MF459655.1	Porcine pegivirus	Porcine	no
MG874672.1	Porcine pegivirus	Porcine	no
MT276210.1	Pegivirus equi	Equine	no
NC_020902.1	Equine pegivirus 1	Equine	no
KC410872.1	Equine pegivirus 1	Equine	no
NC_038435.1	Pegivirus G	Bat	no
NC_038434.1	Pegivirus F	Bat	no
LC602140.1	Rodent pegivirus	Rodent	no
LC602141.1	Rodent pegivirus	Rodent	no
OP589986.1	Bat pegivirus	Bat	no
AF176573.1	Hepatitis C virus	Homo sapiens	no
OQ832071.1	Hepatitis C virus	Homo sapiens	no

Supplementary Table 22. Sequences for *Human blood-associated dicistrovirus* phylogenetic analysis

Accession number/ Sample name	Virus name	Host/Source	This study
OM953859.1	Flumine dicistrovirus 1	River water	no
OQ835731.1	Human blood-associated dicistrovirus	Homo sapiens	no
OR031233.1	Human blood-associated dicistrovirus	Homo sapiens	no
MH370347.1	Bat dicistrovirus	Bat	no
NC_035115.1	Apis dicistrovirus	Bee	no
ON872533.1	Bat faecal associated dicistrovirus 1	Bat	no
OP884011.1	Army ant associated dicistrovirus 3	Army ant	no
OP884017.1	Army ant associated dicistrovirus 7	Army ant	no
OQ715887.1	Wenzhou bat dicistrovirus 6	Bat	no
KY354239.1	Apis dicistrovirus	Bee	no
MH188004.1	Culex dicistrovirus 1	Mosquito	no
MH188005.1	Culex dicistrovirus 2	Mosquito	no
MZ822070.1	Apis dicistrovirus 2	Bee	no
MZ822071.1	Apis dicistrovirus 3	Bee	no
MZ822072.1	Apis dicistrovirus 4	Bee	no
NC_031688.1	Mosquito dicistrovirus	Mosquito	no
K4	Human blood-associated dicistrovirus	Mosquito	yes
K5	Human blood-associated dicistrovirus	Mosquito	yes
MT224137.1	Soybean thrips dicistrovirus 2	Soybean thrips	no
OM953862.1	Flumine dicistrovirus 2	River water	no
OM953865.1	Flumine dicistrovirus 3	River water	no
