# Supplementary material for: Evidence of dengue transmission and a diverse Aedes mosquito virome on the Congo’s Angola border
Source: Nat Commun. 2026 Aug 10;17:9560. doi: 10.1038/s41467-026-76453-z (PMC13550466; doi:10.1038/s41467-026-76453-z)
Supplement: Supplementary file 2 [file 41467_2026_76453_MOESM2_ESM.pdf]

nature portfolio

Peer Review File

Evidence of dengue transmission and a diverse Aedes mosquito virome on the Congo's Angola border

Corresponding Author: Dr Jonathan Parr

This file contains all reviewer reports in order by version, followed by all author rebuttals in order by version.

Version 0:

Reviewer comments:

Reviewer #1

(Remarks to the Author)
The paper by He and colleagues presents a metagenomic study of Aedes mosquito pools from three regions of the Democratic Republic of the Congo (DRC). This is an interesting dataset from a poorly studied region where arboviruses pose a major and growing threat to human health. In this context, the results are important and timely. However, the paper's conclusions are limited by the small sample size and low genome coverage of several viral families, most notably dengue virus.

1) The approaches taken for sequencing and metagenomic analysis are reasonable, and the results yield several expected species further indicating the appropriateness of these choices. I have a few specific questions on this section:
a) There is no mention of thresholds used for filtering hits from KrakenUniq results. Do the authors consider any taxon identified with 1 or more reads to be a positive result? Or are additional cut-offs applied? Given in particular the depth of the sequencing performed here, I would expect some level of filtering would be needed to avoid too many false positive hits. Please can the authors provide more details here.
b) Blast confirmed very few of the taxa identified by KrakenUniq. What do the authors make of this lack of overlap? Did they investigate what other taxa these reads mapped to instead to improve detection/filtering in the future? Relatedly, are the authors surprised they don't see more pathogens in these mosquitoes given the disease burden and known recent cases of CHIKV and YFV?
c) I appreciate the presentation of both genus and family level results (since these can provide different perspectives), however, the authors do not really connect the insights brought by looking at the data in this way. Rather than describing each in turn, which is often repetitive, perhaps focusing on one in more detail and mentioning the other where it adds extra insight would be clearer.

2) The metagenomic sequencing data yields several interesting observations but the presentation of the results is brief and descriptive. Sequencing of additional pools would greatly improve the power of the study to draw more general conclusions about the mosquito virome. If this is not possible, deeper analysis of the existing data would potentially yield richer insights than are currently discussed. In this context:
a) Why were only eight of the 37 pools selected for sequencing? And what was the rationale behind the ones chosen? It is a shame, for instance, that there is only one pool from Malanga which would have permitted more informative comparisons of mosquito viromes between regions, as the single Malanga sample is quite distinct (based on the PCA plot). Could more samples be added?
b) Very little is made of the PCA clustering (Figure S1), though I found this to be among the most interesting results presented. I was surprised that K4 was separating so much from the rest in PC2 given the top families/genera presented in Figure 3 (in which K2 seems most distinct and K4 and K3 look quite similar). Could the authors elaborate on what is driving these signals?
c) Can the authors attempt to account for the pooled nature of their samples, or describe trends seen across pools in more depth. For example, are more viral taxa found in larger pools?

3) The authors conclude their findings show strong evidence of endemic DENV transmission in this part of DRC based on a combination of viral metagenomic and blood meal analysis. While this seems plausible, I had some questions and concerns

especially regarding the phylogenetic analyses performed;
a) What is known about the kinetics of blood meals & persistence of host vs pathogen RNA/DNA? Has it been demonstrated that both would persist for a similar length of time in mosquito bodies such that presence of both in the same sample can reasonably be assumed to come from a common source?
b) Given the emphasis of the paper on the findings around DENV, the poor coverage / assemblies is a major limitation. Could the authors use the recently published tiled amplicon panel, or even just sequencing of their polyprotein gene amplicons to provide better resolution to their phylogenies?
c) I do not feel there is sufficient evidence to conclude on the phylogenetic similarity of these DENV sequences to global samples. In addition to the very short sequences used for the phylogenies, which should be improved, there are very few contextual sequences included on the phylogenetic trees. Indeed, there are no other African DENV4 sequences in figure 4B. The tree topology is also unexpected - several of the DENV4 sequences appear more distinct from the DRC sequences than DENV3 sequences - further challenging the accuracy of the conclusions that can be drawn from the current tree. This is further supported by the weak bootstrap support for many nodes, including those quite basal to the tree. Finally, could the authors explain why they choose a neighbor joining tree here? In general a maximum likelihood tree would be preferred for such an analysis.

4) Regarding presentation of the manuscript, the text was clear but brief and cites relevant publications. I felt the figures could be improved to make them easier to interpret and more concise.
a) In figure 3, the venn diagram is difficult to interpret given the imbalance in the number of pools per site and the barplots contain legends for many families that do not appear. Might a heatmap be an easier way to display this information? This would not require limiting the plot to only the top 3 or 5. A minor aside, in figure 3 legend there are 2 "b" subpanels listed (the second should be "d" I believe).
b) I found the shapes and colors in figure 4 hard to follow. Additionally, as none of the viral database reads are confirmed by blast, I might consider removing the viral database from the approach entirely as it is not clear that it contributes much insight here.
c) For the phylogenetic trees, in addition to my comments above on adding more contextual samples, more details on the exact sequence used would be helpful.
d) Figure 6, I don't find the species icons helpful here and they are hard to see. The sources would also need to be credited.
e) Please credit biorender if it was used for figure making (some of the icons resemble biorender icons)

(Remarks on code availability)
I did not install and run the code, but from reading through it seems complete / corresponds with the analyses presented. While I could not see a readme, the annotation was quite detailed.

Reviewer #2

(Remarks to the Author)
The authors describe their work characterizing the virome of Aedes mosquitoes on the DRC Angola border. I think this is a really interesting study, and a direction that the genomic surveillance field should be moving in. However, I have a few comments and questions that I would like to see addressed before I feel the manuscript is ready for publication:

In the abstract, clarify that this is from 115 total mosquitos spread across 8 pools from 3 sites.
In methods for sample collection - make clear that mosquitos were not separated species specific pools and all Aedes species were lumped together.
Line 132: Give versions and date accessed for the KrakenUniq tool and NCBI-nt and viral databases. These are updated regularly and could give different results.
Line 150: How were nanopore reads trimmed?
Line 164-167: Do you provide a breakdown of Aedes species per site?
Line 190: Clarify if you found 64 total viral genera or if they were 64 unique total genera.
Line 210: When you say long contig, what is your definition? Do you mean full genome? Gene fragment? Is there a cutoff?
Can you provide coverage plots for all of the viruses you call out (DENV, Pegivirus, etc.)? It is difficult to understand the phylogenetic results without seeing how much data is being used to generate these trees.
For the phylogenetic analysis, I recommend doing this in a stepwise manner. To subtype your DENV reads, I would perform the analysis that you did - placing your reads amongst many serotypes to see where they cluster. To understand where it clusters geographically, I would generate a serotype specific tree with more data to understand.
Figure 5 - the colors used for serotype are hard to discern.

(Remarks on code availability)
Both scripts are well annotated. The authors note that due to the size of the dataset, some databases could not be accessed and rather than run in CodeOcean need to be run on your own local machine. Because of accessioning and versioning, this may lead to slightly different results (e.g NCBI nt database is routinely updated as new data becomes available).

Reviewer #3

(Remarks to the Author)
Evidence of dengue virus transmission and a diverse Aedes

mosquito virome on the Democratic Republic of Congo-Angola
border
Running title: Aedes mosquito virome in the DRC
Wenqiao He 1# , Thierry Bobanga 2 , Anne Piantadosi 3,4 , Zachary R. Popkin-Hall 1 , Fabien Vulu 2 ,
Matthew H. Collins 4 , Melchior M. Kashamuka 5 , Antoinette K. Tshefu 5 , Jonathan J. Juliano 1 *,
Jonathan B. Parr

The authors carried out a study on the prevalence of arboviruses in the Democratic Republic of Congo. Malaria is well described in this country, but few studies have been carried out on arboviruses. Mosquitoes were collected in 3 regions of the country. The authors describe the presence of dengue virus in the pools of 115 mosquitoes after metagenomics or targeted sequencing. Other pathogens were also detected.
Major comments:
L95 what was the protocol used to amplify the cytochrome c oxidase?
L97 description of sequencing should not be in this part but in a specific part.
How much RNA input were used to construct libraries?
L110 how much reads were expected?
L111 this paragraph is hard to understand. Do the authors know DENV presence? Please reformulate the paragraph in describing the test not the results
L115 could the authors develop and reformulate the paragraph. It is hard to understand why cytochrome b and 16S allow to understand the blood meal source
Could the authors add the protocol and primers in supplemental data?
Does the RT qPCR detection system discriminate between DENV serotypes or is it a DENV pan?
L127 Could the authors described from where these mosquitoes come from and develop the explanation of their origin. The paragraph should be reformulated
L131 the terms “field mosquito pools” is not hard to understand
L139 the authors speak of reads blasting against humans when they have previously been eliminated
Are the authors talking about reads isolated thanks to kraken? Or are they starting from the pool before kraken?
I don't understand why the authors have double-checked the kraken results with blasts, knowing that to use kraken you need to download the blast database. Furthermore, it is described that kraken was used on an nt database and on a virus database. Can the authors describe the results obtained for each of the 2

L141 Why have the authors aligned contigs and do not realised a mapping of the reads and mapped consensus sequence?
A description of the parameters used to build contigs, read trims and mappings is essential.
L144 phylogenetic trees were obtained from contigs or viral consensus sequences?
L145 mega version and citation

L145 the tree has been made in neighbour joining, it would be better to make it in maximum likelihood. How is the model determined?
Can the authors add the sequences selected to make the tree? How were they selected?
L150 do all the reads were blasted or contigs were first build then blast?
L153 could the authors add a title for the statistical paragraph and differentiate it from mapping one
Could the authors describe the tests used in addition to the program
L162 to facilitate study why authors do not pool the mosquitoes depending their species. This would have made it possible to tell whether one species was more responsible than others for a certain arbovirus.
L171 please add the parameters of filtration in the materials and methods part
L174 “Adapter content was not found in unclassified reads”. what does this sentence mean? Please develop. Adapters were previously removed why they should be find here?

L180: could the authors clarify the cut-off used to say that the families are indeed present and that it is not a sequence conserved in both the host and the virus, a sequence integrated into the genome or an environmental contamination?
Could the authors add a table describing the contigs length and numbers of reads for each viral family
Figure 3A: what does the numbers in the circle mean?
L200: the authors can specify the threshold at which viruses are confirmed and not considered as external contaminants. a table showing the results must be presented in the main figures.
l207 could the authors rephrase this? it says “blast confirmed reads mapping” and later “are also observed in mosquito pools”. I knew that the reads were obtained from mosquito pools. please clarify
l210 why the authors only try to build contigs and not map the reads on the DENV reference find thanks to blast?
in table S5 sample K1 has only one read blasting on DENV? what is the length of this read? how can the authors be sure that it is not a contamination knowing that it comes out negative in RT qPCR?
information on the quantity, length and coverage of genomes is very light and needs to be strengthened and confirmed
I don't understand how there can be a total of 1 read for K1, but per million reads that adds up to 10.3. Accounting for the number of reads is an accounting regardless of the total number of reads unless it's a proportion, in which case a % should be added or 0.10 should be entered.
L230 are the authors sure that this is indeed from rodents, sheep... and that it is not a matter of sequences conserved between several species?
L231: the authors talk about other viruses, but why don't they mention them? they're not a major public issue? why focus on dengue? did they find YFV, ZIKV as described in the abstract?
There is no mention of RT qPCR confirmation in the results, although it is described in the materials and methods.
Figure 5: why didn't you use dengue 4-specific primers for the genome and try to amplify the complete genomes rather than

making a tree with a piece of the 5' UTR? Could the authors try this manipulation?
“Table S6. DENV read counts of other animal and human viruses” Is there an error in the title?
L284 shouldn't the pathogen have been degraded by the mosquito's intestinal system if it came from humans and wasn't “compatible” with the mosquito?
could the authors depool the mosquitoes and test them one by one, which would provide information on the prevalence of the dengue virus at least in the region and the mosquito?
L297: what is the proportion of these virus in the total number of reads . Are they significative?

(Remarks on code availability)

Version 1:

Reviewer comments:

Reviewer #1

(Remarks to the Author)
The authors have addressed all my comments. While it is a shame that more complete sequences could not be added to improve the Dengue classification, I fully appreciate the challenging nature of the samples and thank the authors for all their efforts to generate new data here.

One additional small suggestion - Figures 1 and 2 refer to "Ceco" while the samples are named with a K and the text almost exclusively refers to them as Kimpese City (Ceco only appears once in text). The authors might consider picking just one term and referring to the samples in text and figures consistently throughout.

(Remarks on code availability)
I did not run the code, but confirmed the readme document is now available in the updated GitHub directory.

Reviewer #3

(Remarks to the Author)

(Remarks on code availability)
The manuscript is in progress but still quite difficult to follow.
The emphasis is on viruses whose presence has not been confirmed by RT PCR.

Reviewer #4

(Remarks to the Author)
General comment
In this work, the authors use metagenomics and bloodmeal analysis approaches to investigate the virome and host-vector-virus interactions in Aedes mosquito populations from three distinct areas in the Democratic Republic of Congo, along its border with Angola. They identify viruses from multiple families and in particular, they detect the presence of DENV in a large proportion of the tested pools. This is a rare xenosurveillance initiative, covering an understudied region endemic for multiple arboviruses, likely prone to virus emergence and where sylvatic and urban arbovirus transmission cycles may overlap. The authors make several changes to their manuscript to answer the first round of comments; however, the small sample size, the limited genomic coverage and the weaknesses in the phylogenetic analyses restrict the scope of the results and the conclusions of the study.

Major comments

1. Limitations of the PCA analysis

This aspect has been brought up already but the small sample size and the imbalance of the samples analyzed between study sites is a major limitation of the study as it hinders any meaningful comparison across sites.

2. Relevance and robustness of the contig and subsequent phylogenetic analysis

This part of the study has been brought up by other reviewers already but there are several points that remain of concern to me regarding the analysis of the DENV reads and subsequent phylogenetic analysis.

a. What do the authors mean exactly by phylogenetic analysis of paired reads ? It is still unclear to me from the main text whether any consensus was built from the reads or if the read sequences were directly used for phylogenetic analysis. Please clarify that point in the main text. If an alignment to a reference was performed, which reference was used ? Alignment efficiency can be limited by divergence between the reference and the reads aligned so I suggest selecting the closest known relative (using blast) and then aligning to that reference, that could help building a larger consensus

sequence.

b. Please, provide a plot, map or else specifying what portion of the genome is actually covered by the consensus for each of the sequences analyzed. Also, please specify what portion of the virus genome is covered by the alignment used for phylogenetic tree building. In particular, it is mentioned that part of the reads map to a non-coding part of DENV genome (5' UTR), but tree inference should be performed on coding and non-coding regions separately since they are assumed to evolve under distinct evolution regimens.

c. The phylogenetic analysis appears extremely fragile, reconstructing a tree based on such small sequences (100-200 bp) will result in unreliable phylogenetic placement due to the limited information available. Further, the phylogenetic context provided is extremely poor, and would not provide any relevant context to draw conclusions on the possible origin of the lineages currently circulating in the Kempese area (if any reliable inference was possible, which is unlikely given the size of the sequences analyzed).

d. I suggest removing the pan-serotype analysis of DENV, as more in-depth analysis with serotype-specific phylogenies would be more accurate and useful.

e. I recommend using a threshold of 80 for highlighting nodes in the phylogenetic tree.

3. Absence of investigation of insect-specific viruses

The authors mention detecting several species of insect-specific viruses in several mosquito pools. Why don't they attempt consensus building and phylogenetic analysis of these viruses ? Such data could provide indirect elements to better understand exchanges across the different areas analyzed and e.g. help analyze the surprising clustering results of the PCA.

Minor comments

The introduction is quite brief. Additional context regarding the Kempese region would be useful there.
L54. Please use suitable rather than conducive.
L69-72. Please provide references for this statement.
Methods. Why don't the authors build the tree with iqtree directly following the identification of the best-fit model ?
L186-188. Please provide a more detailed view of how that should be implemented. As evidenced in the present study, the chances of identifying human/animal pathogens in a given location are not that high, so efforts should be put into describing a suitable sampling/surveillance scheme for these activities.
L189-190. Any in the Kempese area ? Or close to it ?
L196-198. Not useful here in my opinion as there is no report of severe dengue cases to discuss in that specific context as far as the discussion goes.
L200-202. Concerning, what about available sequences from neighbouring countries ?
L262-264. Are there important differences between the different locations in terms of elevation, land use patterns, or vegetation ?

(Remarks on code availability)

Version 2:

Reviewer comments:

Reviewer #3

(Remarks to the Author)
I don't understand the sequencing results l 78.
A single sample containing all the pools was sequenced. If that's the case, it should be stated beforehand. Otherwise, if the authors created several sequencing libraries and pooled all the reads, it makes no sense and the analysis will probably have to be redone.
This group is mentioned as l96 "laboratory-uninfected Ae. aegypti mosquito pool", but the group has not been described, including in the mosquito collection description.paragraphaph l96-107 hard to understand, need to be reformulated
l 131: descriptions of kraken or filter conditions should not be included in the text; they should appear before the description of sequencing results.

(Remarks on code availability)

Reviewer #4

(Remarks to the Author)
The authors made several changes to their manuscript following the last round of reviews, addressing most comments. I

have only one minor comment remaining (see below). As mentioned in my previous review, this study has value as it is a rare xenosurveillance initiative, covering an understudied region endemic for multiple arboviruses, likely prone to virus emergence, and where sylvatic and urban arbovirus transmission cycles may overlap. But in spite of the interest of this study, the small sample size and the limited genomic coverage still restrict the scope of the results and the conclusions of the study. For this reason, I do no think this study meets the high standards of this journal.

Minor comment:

l220-222 and 307-308: Please provide values to allow the reader to formally assess what is meant by "highly similar"

(Remarks on code availability)

Version 3:

Reviewer comments:

Reviewer #4

(Remarks to the Author)
The reviewers have addressed all my technical comments in the previous round of reviews. As mentioned in my previous review, in spite of the interest of
this xenomonitoring study covering an understudied region endemic for
multiple arboviruses, the small sample size and the limited genomic coverage still restrict
the scope of the results and the conclusions of the study. For this reason, I do no
think this study meets the high standards of Nature Communications.

(Remarks on code availability)

Reviewer #5

(Remarks to the Author)
MEDIATING REFEREE REPORT
NCOMMS-24-84684C
Nature Communications
Manuscript: "Evidence of dengue virus transmission and a diverse Aedes mosquito virome on the Democratic Republic of Congo-Angola border" by Parr et al.

Overall Assessment
The authors have made genuine and meaningful efforts to address Reviewer #3's three specific concerns. The most substantive issue — confusion about the sequencing design — has been fully and clearly resolved. The remaining two concerns have been substantially but not entirely addressed, and I note minor residual issues for each, as detailed below.
Comment 1: Confusion About the Sequencing Setup

Original concern: The reviewer was unclear whether a single pooled sample had been sequenced or whether multiple libraries had been generated and their reads combined — and noted that the latter approach would require the analysis to be redone.
Authors' response: The authors clarify that each of the eight field mosquito pools, along with the laboratory-uninfected Ae. aegypti control and the water control, was prepared as an individually barcoded sequencing library using unique dual indices (NEBNext Multiplex Oligos for Illumina). These libraries were sequenced together on a single NovaSeq run and subsequently demultiplexed post-hoc. Per-pool read counts are now clearly presented in the revised Figure 3a (ranging from approximately 360 million to 598 million raw reads per pool) and described in Lines 85-91 of the revised manuscript.

Verdict: This concern is fully and satisfactorily addressed. The sequencing design is now unambiguous and methodologically sound.

Comment 2: Missing Description of the Laboratory Control; Paragraph Clarity
Original concern: The reviewer noted that the "laboratory-uninfected Ae. aegypti mosquito pool" had not been described in the mosquito collection section, and that the paragraph covering contamination control (formerly Lines 96-107) was hard to understand and needed reformulation.

Authors' response: The laboratory control is now described in the Methods section (Lines 351-353), where the authors state that 20 uninfected Ae. aegypti mosquitoes (strain NR-48920, obtained from BEI Resources via NIH/NIAID) were pooled to serve as a negative control. The formerly problematic paragraph has been revised (Lines 97-109 in the revised manuscript) to more clearly explain: (i) the purpose of the laboratory and water controls; (ii) how residual reads from these controls were assembled into contaminating contigs; (iii) how those contigs were used to filter reads in field mosquito pools; and (iv) why no reads were ultimately removed from field pools, supported by BLAST evidence showing that overlapping viral genera mapped to distinct genomic regions in field pools compared to controls.

Verdict: This concern is substantially addressed. The description of the laboratory control has been added appropriately, and the logic of the contamination filtering workflow is now considerably clearer. However, the paragraph remains technically dense and may still be challenging for readers unfamiliar with metagenomic workflows. I would encourage the authors to consider one further simplification pass on this paragraph to improve accessibility for a broad readership (if the Editor/Authors share the same feeling).

Comment 3: Bioinformatic Filter Descriptions Should Appear in Methods, Not Results
Original concern: The reviewer requested that descriptions of KrakenUniq and filter conditions (RPM threshold, index-hopping filter) be moved from the Results section to the Methods section.
Authors' response: The authors confirm that filtering conditions are now fully described in the Methods section (Lines 417-422), and that the revised Results section includes only a brief pointer (Lines 137-138) directing readers to the Supplementary Notes for pre-filtering results.

Verdict: This concern is partially addressed. While filtering parameters are now appropriately detailed in the Methods, Lines 105-109 of the revised Results section still contain specific filtering threshold details (RPM <10 threshold and index-hopping filter criteria). The authors' justification for retaining this brief mention — to contextualise the reported numbers — is pragmatic and not unreasonable. Although it does not fully honour the reviewer's original intent to remove such methodological descriptions from the Results text, it does no harm as it provides contect to readers.

Recommendation
The authors have responded constructively to Reviewer #3's comments and the manuscript has been meaningfully improved. The most critical concern regarding the sequencing design has been fully resolved. I recommend minor revisions to address comment 2: i.e. a further clarity pass on the contamination filtering paragraph (Lines 97-109.

(Remarks on code availability)

Open Access This Peer Review File is licensed under a Creative Commons Attribution 4.0 International License, which permits use, sharing, adaptation, distribution and reproduction in any medium or format, as long as you give appropriate credit to the original author(s) and the source, provide a link to the Creative Commons license, and indicate if changes were made.
In cases where reviewers are anonymous, credit should be given to 'Anonymous Referee' and the source.
The images or other third party material in this Peer Review File are included in the article's Creative Commons license, unless indicated otherwise in a credit line to the material. If material is not included in the article's Creative Commons license and your intended use is not permitted by statutory regulation or exceeds the permitted use, you will need to obtain permission directly from the copyright holder.
To view a copy of this license, visit https://creativecommons.org/licenses/by/4.0/

REVIEWER COMMENTS

We thank the reviewers for their helpful comments. Line numbers used in the point-by-point responses below correspond to the "tracked changes" version of the manuscript.

Reviewer #1 (Remarks to the Author):

The paper by He and colleagues presents a metagenomic study of Aedes mosquito pools from three regions of the Democratic Republic of the Congo (DRC). This is an interesting dataset from a poorly studied region where arboviruses pose a major and growing threat to human health. In this context, the results are important and timely. However, the paper's conclusions are limited by the small sample size and low genome coverage of several viral families, most notably dengue virus.

1) The approaches taken for sequencing and metagenomic analysis are reasonable, and the results yield several expected species further indicating the appropriateness of these choices. I have a few specific questions on this section:

a) There is no mention of thresholds used for filtering hits from KrakenUniq results. Do the authors consider any taxon identified with 1 or more reads to be a positive result? Or are additional cut-offs applied? Given in particular the depth of the sequencing performed here, I would expect some level of filtering would be needed to avoid too many false positive hits. Please can the authors provide more details here.

Response: Thank you for these comments. In the revised version, we applied a threshold of 10 reads per million (RPM) to account for sequencing depth, as done by some groups (ex: Wang et al., 2024), when filtering hits from the KrakenUniq results. RPM was calculated using the total number of viral reads per library, as determined by the KrakenUniq nt database. To minimize the impact of index hopping, we considered a virus read count to be a false positive if it accounted for less than 0.1% of the highest read count for that virus within the same sequencing lane (Pan et al., 2023). Details are provided in the Methods section (Lines 487-492). We have retained the KrakenUniq annotation results prior to applying the RPM < 10 threshold and index-hopping filter in the Supplementary Notes (Lines 232-238).

References:

<u>Wang, Y. *et al.* Metagenomic sequencing reveals viral diversity of mosquitoes from Shandong Province, China. *Microbiol Spectr* e0393223 (2024).</u>

<u>Pan, Y.-F. *et al.* Metagenomic analysis of individual mosquito viromes reveals the geographical patterns and drivers of viral diversity. *Nat Ecol Evol* **8**, 947–959 (2024).</u>

b) Blast confirmed very few of the taxa identified by KrakenUniq. What do the authors

make of this lack of overlap? Did they investigate what other taxa these reads mapped to instead to improve detection/filtering in the future? Relatedly, are the authors surprised they don't see more pathogens in these mosquitoes given the disease burden and known recent cases of CHIKV and YFV?

Response: The metagenomic short-read sequencing approach may lack sufficient specificity for species-level annotation, particularly when distinguishing closely related species, which could explain the observed lack of overlap. We have highlighted this limitation in the revised manuscript (Lines 400-403). However, metagenomic sequencing nonetheless provides a culture-independent and unbiased approach to evaluate nucleic acids present in a sample. In the revised version, we reanalyzed the data using the thresholds above to reduce the risk of false positive calls (Lines 487-492). These more stringent filters also resulted in greater overlap between the KrakenUniq and BLAST results. The updated results are now presented in revised Figure 5a.

While the circulation of CHIKV and YFV in the DRC has been documented, several factors may explain their absence in our mosquito pools. First, their transmission is associated with irregular outbreaks in the DRC. Although mosquito collection was conducted during the rainy season—the high transmission period for mosquito-borne diseases— and across multiple sites, our sampling did not comprehensively sample the entire region and was limited to an 8-day period (Lines 416-420 and 431-434). It is therefore possible that we missed circulating arboviruses that peaked at other times of year or geographical regions. We have included this limitation in the revised manuscript (Lines 388-391). In future studies, we plan to collect and sequence additional mosquito pools to obtain a more comprehensive view.

c) I appreciate the presentation of both genus and family level results (since these can provide different perspectives), however, the authors do not really connect the insights brought by looking at the data in this way. Rather than describing each in turn, which is often repetitive, perhaps focusing on one in more detail and mentioning the other where it adds extra insight would be clearer.

Response: Thank you for this suggestion. In the revision, we have moved detailed family level discussion to the Supplementary Notes and focused the main manuscript on the genus-level results and their implications as suggested (Lines 180-231).

2) The metagenomic sequencing data yields several interesting observations but the presentation of the results is brief and descriptive. Sequencing of additional pools would greatly improve the power of the study to draw more general conclusions about the mosquito virome. If this is not possible, deeper analysis of the existing data would potentially yield richer insights than are currently discussed. In this context:
a) Why were only eight of the 37 pools selected for sequencing? And what was the rationale behind the ones chosen? It is a shame, for instance, that there is only one pool from Malanga which would have permitted more informative comparisons of mosquito viromes between regions, as the single Malanga sample is quite distinct (based on the PCA plot). Could more samples be added?

Response: We agree with the reviewer that sequencing more pools would increase the generalizability of our findings and allow for more robust region-level comparisons. However, additional sequencing is not feasible within the scope of the current project, and if undertaken, differences in sample age at the time of processing could bias our results. In this study, we collected a total of 37 mosquito pools (21 from Kimpese city, 6 from Malanga, and 10 from Viaza). To ensure sufficient sequencing depth within a single sequencing run, we randomly selected approximately 20% of the pools from each collection site, resulting in a total of eight pools for metagenomic sequencing (Lines 431-434). We have acknowledged that downsampling was necessary to accommodate sequencing costs in the revised manuscript (Lines 388-391). We are working toward additional mosquito collections and sequencing in our future work to enable more comprehensive analysis of regional differences in the mosquito virome.

Updated PCA results are presented in Figure S3, after reanalyzing the data using more stringent filtering thresholds (Lines 487-492). While the single Malanga sample remains distinct from the mosquito pools collected in Kimpese city and Viaza, geographical clustering is now also observed among the samples from Kimpese city and Viaza. Interestingly, two mosquito pools from Kimpese city differ substantially from the other three pools collected at the same site (Lines 228-231). See further discussion of this finding below.

b) Very little is made of the PCA clustering (Figure S1), though I found this to be among the most interesting results presented. I was surprised that K4 was separating so much from the rest in PC2 given the top families/genera presented in Figure 3 (in which K2 seems most distinct and K4 and K3 look quite similar). Could the authors elaborate on what is driving these signals?

Response: While the single Malanga sample remains distinct from the mosquito pools collected in Kimpese city and Viaza after reanalysis of data using more stringent thresholds, geographical clustering is now observed among the samples from Kimpese city and Viaza as noted above.

To better understand the drivers of the observed clustering, as well as why the two mosquito pools from Kimpese city differ from others, we investigated the top ten viral genera contributing to each principal component in the PCA. These genera are:

PC1	PC2
Certrevirus	Orthophasmavirus
Elunavirus	Orthoflavivirus
Ghunavirus	Cripavirus
Jacunavirus	Mimivirus
Mitovirus	Simplexvirus

Namakavirus	Tombusvirus
Narnavirus	Ourmiavirus
Roskildevirus	Alphamesonivirus
Phasivirus	Jonvirus
Iflavirus	Alphabaculovirus

Viruses within most of these genera are related to insects (*Orthophasmavirus*, *Cripavirus*, *Phasivirus*, *Iflavirus*, *Alphamesonivirus*, *Jonvirus*, and *Alphabaculovirus*), plant (*Tombusvirus* and *Ourmiavirus*), and bacteria (such as *Ghunavirus*, *Jacunavirus*, *Namakavirus*, and *Roskildevirus*). These findings suggest that local ecological and environmental factors—such as elevation, land use patterns, or vegetation—are shaping the virome in mosquitoes from Kimpese city and Viaza. For example, the Malanga pool could be distinct from others owing to its unique highland environment and vegetation. In addition, the variability observed between mosquito pools within Kimpese city may reflect microenvironmental differences or heterogeneous exposure to viral sources within an urban landscape. We have added discussion of these differences in lines 377-384.

c) Can the authors attempt to account for the pooled nature of their samples, or describe trends seen across pools in more depth. For example, are more viral taxa found in larger pools?

Response: We have included the number of mosquitoes in each pool in Figure 2b. We do not observe a relationship between the number of viral taxa and the number of mosquitoes in the field-collected pools (now described in Lines 183-184).

3) The authors conclude their findings show strong evidence of endemic DENV transmission in this part of DRC based on a combination of viral metagenomic and blood meal analysis. While this seems plausible, I had some questions and concerns especially regarding the phylogenetic analyses performed;

a) What is known about the kinetics of blood meals & persistence of host vs pathogen RNA/DNA? Has it been demonstrated that both would persist for a similar length of time in mosquito bodies such that presence of both in the same sample can reasonably be assumed to come from a common source?

Response: Similar windows of detection have been observed for pathogen DNA/RNA and human DNA/RNA in mosquitoes following a blood meal. Based on previous research, complete digestion of a human blood meal typically takes between 36 hours and 3 days, although this duration can vary depending on the mosquito species (Hiroshige et al. 2017; Drummond et al. 2020). The persistence of pathogen RNA or DNA within mosquitoes also varies widely, depending on the pathogen type, strain, viral titer, and mosquito species. Non-arboviral RNA can remain detectable in the mosquito midgut for 24 to 36 hours, whereas

DENV RNA is typically undetectable after 24 hours unless the virus successfully replicates in mosquito tissues—at which point RNA levels may rise again between 100 and 200 hours post-blood meal.

Given these kinetics, the co-detection of human DNA and DENV RNA in the same mosquito pools suggests a common source, likely a viremic human host. The presence of DENV RNA in two mosquito pools (K4 and V3), both of which appear to have fed exclusively on human blood, further supports the idea that DENV is actively circulating in these communities. We have added this discussion to the revised manuscript (Lines 336-351).

Separately, we identified and corrected an error in the manuscript text describing the number of bloodmeals from rodents and canines (lines 279-281).

References:

Hiroshige Y. *et al.* A human genotyping trial to estimate the post-feeding time from mosquito blood meals. *PLoS One* (2017).

Drummond C. *et al*. Stability and detection of nucleic acid from viruses and hosts in controlled mosquito blood feeds. *PLoS One* (2020)

b) Given the emphasis of the paper on the findings around DENV, the poor coverage / assemblies is a major limitation. Could the authors use the recently published tiled amplicon panel, or even just sequencing of their polyprotein gene amplicons to provide better resolution to their phylogenies?

Response: Thank you for the comment. We agree with the reviewer that limited coverage of DENV is an important limitation of our study. To improve the resolution of our phylogenetic analysis, we attempted but failed to amplify DENV polyprotein gene fragments and regions adjacent to the detected DENV reads using published assays (Fraenkel et al. 2024; Meng et al. 2023; Vogels et al. 2024). We also designed several primer sets targeting DENV-2 and DENV-4 that perform well on standard/control RNA but did not amplify their targets in our pools.

These efforts likely failed due to low DENV RNA concentration and RNA degradation in our samples (Lines 258-261). RNA fragment sizes within the mosquito pools were short (across pools the Agilent TapeStation median peak was 176nt, IQR 115 - 178). Such short fragment lengths are likely to remain a fundamental limitation of this type of metagenomic sequencing approach due to RNA degradation within the mosquito (see comments above). This limitation is highlighted in the revised manuscript (Lines 391-397). However, successful amplification of a distinct DENV target using real-time PCR in the samples with the highest DENV read counts increases our confidence in our DENV calls, as do reports of positive DENV rapid test results observed during a recent, unpublished non-malarial febrile illness study in Kimpese.

c) I do not feel there is sufficient evidence to conclude on the phylogenetic similarity of these DENV sequences to global samples. In addition to the very short sequences

used for the phylogenies, which should be improved, there are very few contextual sequences included on the phylogenetic trees. Indeed, there are no other African DENV4 sequences in figure 4B. The tree topology is also unexpected - several of the DENV4 sequences appear more distinct from the DRC sequences than DENV3 sequences - further challenging the accuracy of the conclusions that can be drawn from the current tree. This is further supported by the weak bootstrap support for many nodes, including those quite basal to the tree. Finally, could the authors explain why they choose a neighbor joining tree here? In general a maximum likelihood tree would be preferred for such an analysis.

Response: In the revised version, we used IQ-TREE to identify the best-fit available model and regenerated maximum likelihood (ML) trees using the best model with RAxML-NG (Lines 507-509). We also added more reference sequences from GenBank to improve the contextual representation as suggested, including additional African DENV-4 sequences where available. To select published sequences, we randomly selected sequences available in the NCBI Virus, RefSeq, as well as GenBank databases that were >10,000 bp, represented different serotypes, and originated from different geographic regions. Additionally, we included reference sequences with known genotype information from two published studies (Arshi I. et al., 2023; Surya P.Y. et al., 2021). Details about publicly available sequences included in the revised trees are provided in Supplementary Tables S10-S15.

We note that DENV-4 reads detected in our study also mapped to Wenzhou sobemo-like virus 4 sequences using BLAST. However, the similarity was lower (nucleotide identity ≤ 96.67% and higher e-values) compared to the DENV-4 published sequence (nucleotide identity = 97.8%) (Lines 255-258 and 261-262). Updated ML trees (Figures 5b and 6) continue to cluster our reads with DENV-2 and DENV-4 sequences, providing support for BLAST classifications. However, we acknowledge that the bootstrap support for several nodes remains weak and that the limited sequence coverage in our study constrains the resolution of our phylogenetic analysis, as well as our ability to comment confidently on relatedness to sequences from other geographies (Lines 263-264).

References:

Arshi I. *et al.* Global and local evolutionary dynamics of Dengue virus serotypes 1, 3, and 4. *Epidemiol Infect* (2023).

Surya P.Y. *et al*. Evolution, heterogeneity and global dispersal of cosmopolitan genotype of Dengue virus type 2. *Sci Rep* (2021)

4) Regarding presentation of the manuscript, the text was clear but brief and cites relevant publications. I felt the figures could be improved to make them easier to interpret and more concise.
a) In figure 3, the venn diagram is difficult to interpret given the imbalance in the number of pools per site and the barplots contain legends for many families that do not appear. Might a heatmap be an easier way to display this information? This would

not require limiting the plot to only the top 3 or 5. A minor aside, in figure 3 legend there are 2 “b” subpanels listed (the second should be “d” I believe).

Response: Thank you for the suggestion. We have replaced the Venn diagrams with heatmaps in the revised manuscript (Figure 4). The relative abundance bar plots have been moved to Figure S2.

b) I found the shapes and colors in figure 4 hard to follow. Additionally, as none of the viral database reads are confirmed by blast, I might consider removing the viral database from the approach entirely as it is not clear that it contributes much insight here.

Response: We have removed the results from the viral database as suggested and used distinct colors to show different annotation results (Figure 5a).

c) For the phylogenetic trees, in addition to my comments above on adding more contextual samples, more details on the exact sequence used would be helpful.

Response: We have added more reference sequences for the phylogenetic analysis as described above. Additionally, we report the coordinates of our reads based on the most similar sequences (Lines 253-258).

d) Figure 6, I don’t find the species icons helpful here and they are hard to see. The sources would also need to be credited.

Response: We have removed the species icons in Figure 8 as suggested.

e) Please credit biorender if it was used for figure making (some of the icons resemble biorender icons)

Response: Thanks for catching this. Figure 1 was created using BioRender, and proper credit has been added to the figure legend.

Reviewer #1 (Remarks on code availability):

I did not install and run the code, but from reading through it seems complete / corresponds with the analyses presented. While I could not see a readme, the annotation was quite detailed.

Response: Thank you for your comment. The updated version of our code is available on GitHub, which includes a detailed Readme file (https://github.com/IDEELResearch/DRCAedesSeq).

Reviewer #2 (Remarks to the Author):

The authors describe their work characterizing the virome of Aedes mosquitoes on the DRC Angola border. I think this is a really interesting study, and a direction that the genomic surveillance field should be moving in. However, I have a few comments and questions that I would like to see addressed before I feel the manuscript is ready for publication:

In the abstract, clarify that this is from 115 total mosquitos spread across 8 pools from 3 sites.

Response: We have added this information to the revised abstract as requested (Lines 32-33 and 431-433).

In methods for sample collection - make clear that mosquitos were not separated species specific pools and all Aedes species were lumped together.

Response: We have added this information to the Methods section (Lines 424), clarifying that mosquitoes were not separated by species and that *Aedes* species were pooled together.

Line 132: Give versions and date accessed for the KrakenUniq tool and NCBI-nt and viral databases. These are updated regularly and could give different results.

Response: We used KrakenUniq version 1.0.4. The NCBI nt database used for the analysis was downloaded on July 31, 2023. These details have been added to the Methods section (Lines 483-485). In the revised version, we removed the results generated based on the NCBI viral database in response to Reviewer 1's recommendation.

Line 150: How were nanopore reads trimmed?

Response: We trimmed barcodes and adapters from nanopore reads using Guppy-barcoder (Line 512-514) with default parameters, including the options --enable_trim_barcodes and --trim_adapters. The code for analysis is available on GitHub (https://github.com/IDEELResearch/DRCAedesSeq).

Line 164-167: Do you provide a breakdown of Aedes species per site?

Response: We have included the number of each *Aedes* species identified morphologically at each site in Table S1.

Line 190: Clarify if you found 64 total viral genera or if they were 64 unique total genera.

Response: In response to Reviewer 1's comments, we applied a more stringent threshold of 10 RPM as well as an index-hopping filter to hits from KrakenUniq (Lines 487-492). After filtering, we found 28 unique total viral genera (Lines 204-208). We have retained results obtained before applying the 10 RPM and index-hopping filter in the Supplementary Notes (64 unique total viral genera, as correctly noted).

Line 210: When you say long contig, what is your definition? Do you mean full genome? Gene fragment? Is there a cutoff?

Response: Apologies for the confusion. We were referring to contigs longer than individual DENV reads. We failed to assemble contigs spanning multiple individual DENV reads. This language has been clarified in the revised manuscript (Line 250-251).

Can you provide coverage plots for all of the viruses you call out (DENV, Pegivirus, etc.)? It is difficult to understand the phylogenetic results without seeing how much data is being used to generate these trees.

Response: In the revised analysis, we applied published thresholds to minimize false positives in the KrakenUniq annotations as noted above. After reanalyzing the data, we detected reads mapping to DENV and *bat fecal-associated dicistrovirus 4*. We have added the requested coverage information for these viruses in the Results section (Lines 253-258 and 265–269) and in Tables S7-S8.

The phylogenetic analysis has also been updated accordingly (Figures 5b, 6, and 7). Unfiltered KrakenUniq results are presented in the Supplementary Notes, and phylogenetic and coverage data for *human pegivirus* and *human blood-associated dicistrovirus* are now included as well.

For the phylogenetic analysis, I recommend doing this in a stepwise manner. To subtype your DENV reads, I would perform the analysis that you did - placing your reads amongst many serotypes to see where they cluster. To understand where it clusters geographically, I would generate a serotype specific tree with more data to understand.

Response: Thank you for the suggestion. In the revised manuscript, we included additional reference sequences and generated a comprehensive phylogenetic tree encompassing all four DENV serotypes (Figure 5b), followed by serotype-specific trees for DENV-2 and DENV-4 (Figure 6) as suggested to enhance resolution and geographic context.

Figure 5 - the colors used for serotype are hard to discern.
Response: We have revised the color scheme in Figures 5b and 6 to enhance clarity and make it easier to distinguish DENV serotypes.

Reviewer #2 (Remarks on code availability):

Both scripts are well annotated. The authors note that due to the size of the dataset, some databases could not be accessed and rather than run in CodeOcean need to be run on your own local machine. Because of accessioning and versioning, this may lead to slightly different results (e.g NCBI nt database is routinely updated as new data becomes available).

Response: Thank you for noting this. To ensure reproducibility, we have included the versions of all packages and software used, along with the download date (July 31, 2023) for the NCBI nt database, in the revised Methods – Data Analysis section (Lines 470–523). The updated code is available on GitHub (https://github.com/IDEELResearch/DRCAedesSeq).

Reviewer #3 (Remarks to the Author):

Evidence of dengue virus transmission and a diverse Aedes mosquito virome on the Democratic Republic of Congo-Angola border
Running title: Aedes mosquito virome in the DRC
Wenqiao He 1# , Thierry Bobanga 2 , Anne Piantadosi 3,4 , Zachary R. Popkin-Hall 1 , Fabien Vulu 2 ,
Matthew H. Collins 4 , Melchior M. Kashamuka 5 , Antoinette K. Tshefu 5 , Jonathan J. Juliano 1 *,
Jonathan B. Parr

The authors carried out a study on the prevalence of arboviruses in the Democratic Republic of Congo. Malaria is well described in this country, but few studies have been carried out on arboviruses. Mosquitoes were collected in 3 regions of the country. The authors describe the presence of dengue virus in the pools of 115 mosquitoes after metagenomics or targeted sequencing. Other pathogens were also detected.

Major comments:
L95 what was the protocol used to amplify the cytochrome c oxidase?

Response: Thank you for catching this omission. We used primers described in references 50 and 52 (lines 442-445) in the cytochrome *c* oxidase amplification protocol in the revised manuscript (Lines 442-445). We have added a supplementary table listing all primers and reaction conditions (Table S9).

L97 description of sequencing should not be in this part but in a specific part.
How much RNA input were used to construct libraries?

Response: In this study, we conducted two sequencing runs: Nanopore sequencing for mosquito species identification and blood meal analysis, and Illumina NovaSeq sequencing for viral metagenomic analysis. To improve clarity, we have moved the sequencing

description to a dedicated section immediately following the library construction details (Lines 451-453 and 462-465).

For Nanopore library preparation, we pooled PCR amplicons from each mosquito pool in equimolar amounts and used 68 ng of total DNA per pool as input for library construction (Lines 449-451).

L110 how much reads were expected?

Response: We used one lane of the NovaSeq S4 platform, which yields approximately 4–5 billion reads in total (corresponding to 2–2.5 billion clusters). We have added this information to lines 465.

L111 this paragraph is hard to understand. Do the authors know DENV presence? Please reformulate the paragraph in describing the test not the results

Response: We use the term "confirmation" because the real-time PCR assay was undertaken after analysis of metagenomic sequencing data in order to confirm DENV calls. We have revised this section in the manuscript to make this clearer (Lines 466-469).

L115 could the authors develop and reformulate the paragraph. It is hard to understand why cytochrome b and 16S allow to understand the blood meal source Could the authors add the protocol and primers in supplemental data?

Response: Previous studies have employed various approaches for mosquito blood meal analysis, including cytological, serological, and DNA-based methods. Among DNA-based methods, mitochondrial genes such as cytochrome b and 16S rRNA are commonly used due to their ability to provide species-specific resolution for identifying vertebrate hosts. We included the references in the revised manuscript (Lines 445-449) and listed the primer sets in new Table S9.

Does the RT qPCR detection system discriminate between DENV serotypes or is it a DENV pan?

Response: It is a pan-DENV assay. We have clarified this in the revised manuscript (Lines 467-469).

L127 Could the authors described from where these mosquitoes come from and develop the explanation of their origin. The paragraph should be reformulated

Response: Uninfected *Aedes aegypti* mosquitoes (NR-48920) were obtained from the NIH/NIAID Filariasis Research Reagent Resource Center through BEI Resources. Twenty of these mosquitoes were randomly selected and pooled to serve as the laboratory uninfected *Ae. aegypti* control pool. We included the details in Methods (Lines 427-429) and Acknowledgements (Lines 526-528).

L131 the terms “field mosquito pools” is not hard to understand

Response: We have removed the phrase "field mosquito pools" to avoid redundancy (Lines 483-485).

L139 the authors speak of reads blasting against humans when they have previously been eliminated

Are the authors talking about reads isolated thanks to kraken? Or are they starting from the pool before kraken?

Response: We apologize for any confusion. In our analysis, we used BLAST to identify reads mapping to both confirmed and potential human pathogens, as well as known and suspected animal pathogens. This clarification has been incorporated into the revised manuscript (Lines 498-499).

I don't understand why the authors have double-checked the kraken results with blasts, knowing that to use kraken you need to download the blast database. Furthermore, it is described that kraken was used on an nt database and on a virus database. Can the authors describe the results obtained for each of the 2

Response: We appreciate the reviewer's question regarding the use of both KrakenUniq and BLAST for taxonomic classification. While both tools can utilize the same reference databases, their underlying algorithms differ significantly: KrakenUniq relies on exact k-mer matching for rapid classification, whereas BLAST employs alignment-based methods and assigns reads to the lowest common ancestor (LCA). By combining these approaches, we aimed to improve confidence in pathogen identification (Lines 498-499).

The majority of our results were based on the nt database. In response to reviewer 1’s suggestion, we have removed the KrakenUniq results based on the viral database from the revised manuscript.

L141 Why have the authors aligned contigs and do not realised a mapping of the reads and mapped consensus sequence?
A description of the parameters used to build contigs, read trims and mappings is essential.

Response: Thank you for your suggestion - the revised manuscript includes these parameters. Standard default parameters were used for read trimming and contig assembly (Lines 472-475 and 499-501). However, we were unable to generate contigs for DENV or bat fecal-associated dicistrovirus 4 due to insufficient read coverage (Lines 250-251 and 269-270). This is due in part to short RNA fragments in the mosquito pools (see response to Reviewer 1). All phylogenetic analyses were performed based on consensus sequences generated from merged paired-end reads with alignment conducted by MAFFT (Lines 501-

506). The updated code is available at github: https://github.com/IDEELResearch/DRCAedesSeq.

L144 phylogenetic trees were obtained from contigs or viral consensus sequences?

Response: Unfortunately, we were unable to generate contigs for DENV or bat fecal-associated dicistrovirus 4 as noted above (Lines 250-251 and 269-270). All phylogenetic analyses were performed based on consensus sequences generated from aligned, merged paired-end reads (Lines 501-506, 263-264 and 269-270).

L145 mega version and citation

Response: In the revised manuscript, all sequence alignments were performed using MAFFT (version 7.490) with default parameters (Line 506).

L145 the tree has been made in neighbour joining, it would be better to make it in maximum likelihood. How is the model determined?

Can the authors add the sequences selected to make the tree? How were they selected?

Response: Thank you for these questions, which are aligned with those from the other reviewers. In the revised analysis, we selected a broader set of reference sequences representing diverse genotypes/serotypes from multiple geographic regions and time periods to provide comprehensive phylogenetic context (Lines 501-506). Accession numbers are included in Tables S10-S15 and also provided in GitHub (https://github.com/IDEELResearch/DRCAedesSeq).

For phylogenetic reconstruction in the revised manuscript, we first determined the optimal substitution model using IQ-TREE (v2.4.0), then generated maximum likelihood trees with RAxML-NG (v1.2.2) under the selected best-fit available model. These methodological details are described in the revised manuscript (Lines 507-509).

L150 do all the reads were blasted or contigs were first build then blast?

Response: For the Nanopore sequencing data, we did not assemble contigs. Instead, we directly performed BLAST on all reads with a Phred quality score ≥ 6 (Lines 514-515).

L153 could the authors add a title for the statistical paragraph and differentiate it from mapping one
Could the authors describe the tests used in addition to the program

Response: We did not perform hypothesis testing as a part of the analysis, so a statistical analysis paragraph was not indicated. However, we did use a suite of different tools to process, analyze, and visualize data generated during the study. We have consolidated this

information as well as version numbers into a single, concluding methods paragraph (Lines 516-523).

L162 to facilitate study why authors do not pool the mosquitoes depending their species. This would have made it possible to tell whether one species was more responsible than others for a certain arbovirus.

Response: We agree that pooling mosquitoes by species could provide insights into species-specific arbovirus associations. However, in our current study, mosquitoes were pooled based on sample collection sites rather than species. We have clarified this in the Methods section (Lines 424) and acknowledged this limitation in the revised manuscript (Lines 385-386).

L171 please add the parameters of filtration in the materials and methods part

Response: We used the “MINLEN:36” parameter in Trimmomatic to filter out short reads (<36 bp) (Lines 474-475). The updated code is available at https://github.com/IDEELResearch/DRCAedesSeq.

L174 “Adapter content was not found in unclassified reads”. what does this sentence mean? Please develop. Adapters were previously removed why they should be find here?

Response: We included this line to ensure clarity that unclassified reads weren’t simply residual adapter sequences. Adapter sequences were successfully removed from downstream analysis. To avoid confusion, we have removed this sentence from the revised manuscript (Line 178).

L180: could the authors clarify the cut-off used to say that the families are indeed present and that it is not a sequence conserved in both the host and the virus, a sequence integrated into the genome or an environmental contamination?

Response: To control for potential confounding factors including host sequence similarity and environmental contamination, we included an uninfected *Aedes aegypti* mosquito pool and a water control pool in our experimental design (Lines 427-429 and 435-439). During bioinformatic analysis, reads mapping to *Homo sapiens*, *Aedes aegypti*, *Aedes albopictus*, and *Aedes simpsoni* were removed to eliminate host-derived sequences (Lines 475-478). Any remaining reads mapping to the contigs or identified as viral genera by KrakenUniq in uninfected *Aedes aegypti* mosquito pool or the water control pool were further removed (Lines 479-483 and 450-452).

In the revised version, we also applied a threshold of 10 RPM and an index-hopping filter as described in response to Reviewer 1’s comments above (Lines 487-492).

Could the authors add a table describing the contigs length and numbers of reads for each viral family.

Response: Thank you for the suggestion. In the revised manuscript, we have included heatmaps to display the reads per million (RPM) for each viral family/genus (Figure 4, with raw data and code available at https://github.com/IDEELResearch/DRCAedesSeq). Regarding contig assembly and assignments, we focused specifically on BLAST-confirmed reads mapping to pathogens and potential pathogens (Lines 499-501). However, we were unable to generate contigs for DENV or *bat faecal-associated dicistrovirus 4* due to insufficient breadth of coverage as outlined above (Lines 250-251 and 269-270).

Figure 3A: what does the numbers in the circle mean?

Response: The Venn diagrams have been replaced by heatmaps in the revised manuscript (Figure 4).

L200: the authors can specify the threshold at which viruses are confirmed and not considered as external contaminants. a table showing the results must be presented in the main figures.

Response: We have moved the supplementary table showing read counts into the main manuscript as requested (Figure 3). In addition to the methods outlined above to filter potential contaminants during bioinformatic analysis, we also employed more stringent analysis thresholds in the revised manuscript that take sequencing depth into account as outlined in our response to other comments above.

l207 could the authors rephrase this? it says “blast confirmed reads mapping” and later “are also observed in mosquito pools”. I knew that the reads were obtained from mosquito pools. please clarify

Response: We have revised this sentence for clarity (Lines 247-250).

l210 why the authors only try to build contigs and not map the reads on the DENV reference find thanks to blast?

Response: We aligned merged paired-end reads to an expanded set of reference sequences in the revised manuscript (Figure 5b). We further confirmed DENV types using BLAST, which confirmed high similarity to DENV-2 and DENV-4 references (Lines 253-258).

in table S5 sample K1 has only one read blasting on DENV? what is the length of this read? how can the authors be sure that it is not a contamination knowing that it comes out negative in RT qPCR?

information on the quantity, length and coverage of genomes is very light and needs to be strengthened and confirmed.

I don't understand how there can be a total of 1 read for K1, but per million reads that adds up to 10.3. Accounting for the number of reads is an accounting regardless of the total number of reads unless it's a proportion, in which case a % should be added or 0.10 should be entered.

Response: Thank you for these comments and suggestions. In addition to moving the table showing read counts into the main manuscript, we have included a supplementary table with more details about the DENV reads, including the length and coordinates of merged paired-end reads from each DENV-positive pool. The DENV reads obtained in our study were short (~100bp, Table S7), which limits the precision of our phylogenetic analyses and is an acknowledged limitation of our approach.

Regarding pool K1, the 90 bp read showed high similarity to the DENV-4 strain 2013JH285 (MG601754.1) and clustered with other DENV-4 genotype I sequences in phylogenetic analysis. We calculated RPM (Reads Per Million) based on the number of viral reads, not total sequencing reads (Lines 487-490). In pool K1, there was 1 DENV read out of 96,956 total viral reads (Figure 3a), which corresponds to approximately 10.3 RPM ($1 \div 96{,}956 \times 1{,}000{,}000 \approx 10.3$). Although the absolute number of DENV reads was low, the total number of viral reads in that pool was also relatively low, which results in a higher RPM value. We hope that the additional detail provided in Table S7 will help avoid confusion.

We expected to observe some inconsistency between the metagenomic and real-time PCR results owing to the detection limits of the qPCR assay, which are as follows (copies/μL): DENV-1, 11.7; DENV-2, 13.5; DENV-3, 4.1; DENV-4, 10.5. The absence of a positive qPCR signal for sample K1 and other pools with lower DENV read counts suggests that the DENV RNA level was below the assay's detection threshold. Consistency between sequencing and real-time PCR among samples with higher DENV read counts provides support for the validity of our calls. DENV RNA was confirmed by qPCR in the three mosquito pools that had the highest DENV read counts in the metagenomic analysis (Lines 274-277).

L230 are the authors sure that this is indeed from rodents, sheep... and that it is not a matter of sequences conserved between several species?

Response: We used two established markers—cytochrome b (*cytB*) and 16S ribosomal RNA—for mosquito blood meal analysis. These assays were selected based on their broad taxonomic coverage and species-specific resolution and have been widely used for species identification (Lines 445-449).

References:

Meyer, R., Höfelein, C., Lüthy, J. & Candrian, U. Polymerase chain reaction-restriction fragment length polymorphism analysis: a simple method for species identification in food. J. AOAC Int. 78, 1542–1551 (1995).

Taylor, P. G. Reproducibility of ancient DNA sequences from extinct Pleistocene fauna. Mol. Biol. Evol. 13, 283–285 (1996).

L231: the authors talk about other viruses, but why don't they mention them? they're not a major public issue? why focus on dengue? did they find YFV, ZIKV as described in the abstract?

Response: We agree that one advantage of metagenomic sequencing is that it enables consideration of numerous pathogens and potential pathogens. Although outbreaks of YFV have occurred in the DRC and ZIKV is known to circulate, we did not detect any reads mapping to these viruses in the selected mosquito pools. Broader sampling across more geographical regions and time points would increase our ability to detect transmission of these and other viruses. We focused our discussion on DENV because it was identified across a majority of mosquito pools (Lines 251-253). We have added more discussion on *bat faecal-associated dicistrovirus 4*-a non-arbovirus (Lines 352-361), and included some discussion of other viruses in the Results and Discussion sections (Lines 239-245 and 371-376).

There is no mention of RT qPCR confirmation in the results, although it is described in the materials and methods.

Response: These results are included in lines 274-277 and Table S7. The pan-DENV real-time PCR assay confirmed the presence of DENV RNA in the three mosquito pools with the highest DENV read counts identified through metagenomic analysis.

Figure 5: why didn't you use dengue 4-specific primers for the genome and try to amplify the complete genomes rather than making a tree with a piece of the 5' UTR? Could the authors try this manipulation?

Response: This is a good suggestion. To improve the resolution of our phylogenetic analysis, we attempted but failed to amplify additional loci using published and custom primers. This is likely due to RNA degradation within the mosquito pools (see fragment length discussion in response to reviewer 1 above) and acknowledged as a limitation in the revised manuscript (Lines 258-261 and 391-397).

“Table S6. DENV read counts of other animal and human viruses” Is there an error in the title?

Response: Thank you for catching this typo. We have removed the term “DENV”.

L284 shouldn't the pathogen have been degraded by the mosquito's intestinal system if it came from humans and wasn't “compatible” with the mosquito?

Response: In this study, we used whole mosquitoes for homogenization and RNA extraction, which means sequences could be generated from both recently ingested pathogens and those that are actively replicating within the mosquito. *Bat faecal associated dicistrovirus 4* is not a mosquito-borne virus, but was probably detectable because the mosquito had not yet

had time to fully digest ingested pathogen nucleic acids. Grubaugh et al. demonstrated that viral RNA remains detectable in the mosquito midgut within a field-relevant time-frame, for example, WNV RNA was detectable for up to 18 hours after blood feeding, and HIV-1 RNA for up to 24 hours. Therefore, it is possible that the detection of viral RNA from non-arboviruses in our study was due to incomplete degradation by the mosquito digestive system at the time of sampling (Lines 339-343 and 353-354).

References:

Grubaugh et al. Xenosurveillance: A Novel Mosquito-Based Approach for Examining the Human-Pathogen Landscape. *PLoS Negl Trop Dis* (2015).

could the authors depool the mosquitoes and test them one by one, which would provide information on the prevalence of the dengue virus at least in the region and the mosquito?

Response: We agree that sequencing individual mosquitoes would provide valuable insights into DENV transmission at both the species and regional levels. Because mosquitoes were homogenized and processed as pooled samples in this study, it is impossible to retrospectively determine individual-level infection status. We acknowledge this as a limitation in the revised manuscript (Lines 397-399). We are working toward future studies in the DRC involving collection of pooled and individual mosquitoes to allow this type of analysis.

L297: what is the proportion of these virus in the total number of reads . Are they significative?

Response: Thank you for your comment. These results are now presented in Figure 3b.

REVIEWER COMMENTS

We thank the reviewers for their helpful comments. Line numbers used in the point-by-point responses below correspond to the "tracked changes" version of the manuscript.

Reviewer #1 (Remarks to the Author):

The authors have addressed all my comments. While it is a shame that more complete sequences could not be added to improve the Dengue classification, I fully appreciate the challenging nature of the samples and thank the authors for all their efforts to generate new data here.

One additional small suggestion - Figures 1 and 2 refer to "Ceco" while the samples are named with a K and the text almost exclusively refers to them as Kimpese City (Ceco only appears once in text). The authors might consider picking just one term and referring to the samples in text and figures consistently throughout.

Response: Thank you for these comments. In the revised version, we consistently use "Kimpese City" throughout the manuscript. We have also revised Figures 1 and 2.

Reviewer #1 (Remarks on code availability):

I did not run the code, but confirmed the readme document is now available in the updated GitHub directory.

Response: Thank you for your comment. We have updated the GitHub to reflect revisions made in response to Reviewer #4's comments below.

Reviewer #3 (Remarks on code availability):

The manuscript is in progress but still quite difficult to follow.

The emphasis is on viruses whose presence has not been confirmed by RT PCR.

Response: We believe the revised manuscript is strengthened by reviewer comments, and we hope easier to follow. A major advantage of the unbiased metagenomic sequencing approach we employed is that it can detect viruses not known or expected to be in circulation. We used RT-PCR to confirm dengue virus infections identified in our pools (Lines 431-433). Targeted RT-

PCR assays are useful for confirming such metagenomic findings; however, performing distinct RT-PCR assays for all detected viruses is beyond the scope of this work.

To minimize false positives, all metagenomic calls were supported by stringent bioinformatic filtering (Lines 439-447 and 451-456). In addition, we performed manual inspection of read alignments for dengue virus and other known and putative human and animal viruses (Lines 462-463, Figure 5).

Reviewer #4 (Remarks to the Author):

General comment

In this work, the authors use metagenomics and bloodmeal analysis approaches to investigate the virome and host-vector-virus interactions in Aedes mosquito populations from three distinct areas in the Democratic Republic of Congo, along its border with Angola. They identify viruses from multiple families and in particular, they detect the presence of DENV in a large proportion of the tested pools. This is a rare xenosurveillance initiative, covering an understudied region endemic for multiple arboviruses, likely prone to virus emergence and where sylvatic and urban arbovirus transmission cycles may overlap. The authors make several changes to their manuscript to answer the first round of comments; however, the small sample size, the limited genomic coverage and the weaknesses in the phylogenetic analyses restrict the scope of the results and the conclusions of the study.

Major comments

1. Limitations of the PCA analysis

This aspect has been brought up already but the small sample size and the imbalance of the samples analyzed between study sites is a major limitation of the study as it hinders any meaningful comparison across sites.

Response: We agree with the reviewer’s comment. Although the randomly selected pools included in this study represent approximately 20% of the total mosquito pools from each region, the overall number of mosquito pools remains limited. Accordingly, PCA was used as an exploratory tool to visualize variation in metagenomic profiles rather than to infer statistically meaningful differences between sites. We have clarified this point and explicitly acknowledged this limitation in the revised manuscript (Lines 130–133 and 335-338).

2. Relevance and robustness of the contig and subsequent phylogenetic analysis

This part of the study has been brought up by other reviewers already but there are several points that remain of concern to me regarding the analysis of the DENV reads and subsequent phylogenetic analysis.

a. What do the authors mean exactly by phylogenetic analysis of paired reads ? It is still unclear to me from the main text whether any consensus was built from the reads or if the read sequences were directly used for phylogenetic analysis. Please clarify that point in the main text. If an alignment to a reference was performed, which reference was used ? Alignment efficiency can be limited by divergence between the reference and the reads aligned so I suggest selecting the closest known relative (using blast) and then aligning to that reference, that could help building a larger consensus sequence.

Response: We agree with the reviewer that our DENV phylogenetic analyses were limited by the length of sequences analyzed. We had previously highlighted this as a limitation of our approach and provided reference sequences/coordinates for transparency; however, we have now chosen to remove these phylogenetic analyses from the revised manuscript completely.

We have replaced them with new analyses of several insect-specific viruses (see response below) for which we could assemble longer contigs for higher confidence phylogenies. Phylogenetic analyses of these and other viruses included in this study were performed using consensus sequences generated from merged paired-end reads, as described in the revised manuscript. Specifically, the closest known relatives were first identified by BLAST and used as references for read alignment to maximize alignment efficiency (Lines 466–468).

b. Please, provide a plot, map or else specifying what portion of the genome is actually covered by the consensus for each of the sequences analyzed. Also, please specify what portion of the virus genome is covered by the alignment used for phylogenetic tree building. In particular, it is mentioned that part of the reads map to a non-coding part of DENV genome (5' UTR), but tree inference should be performed on coding and non-coding regions separately since they are assumed to evolve under distinct evolution regimens.

Response: Thank you for the comment. The portion of the DENV genome covered by the alignment is provided in Table S6. As noted above, we have removed DENV phylogenetic analyses from the revised manuscript.

For phylogenetic analyses of other viruses included in this study, we now provide visualization of genomic regions assessed as requested. See analyses of *bat faecal–associated dicistrovirus 4* (Lines 184–187), *Aedes flavivirus* (Figure S4a), *Wenzhou sobemo-like virus 4* (Figure S5a),

Guangzhou sobemo-like virus (Figure S6a), *Sichuan mosquito sobemo-like virus* (Figure S7a), and *Hubei mosquito virus 2* (Lines 214–218).

c. The phylogenetic analysis appears extremely fragile, reconstructing a tree based on such small sequences (100-200 bp) will result in unreliable phylogenetic placement due to the limited information available. Further, the phylogenetic context provided is extremely poor, and would not provide any relevant context to draw conclusions on the possible origin of the lineages currently circulating in the Kempese area (if any reliable inference was possible, which is unlikely given the size of the sequences analyzed).

Response: See responses above. We have removed the DENV phylogenetic analysis and instead focused our discussion of DENV on its presence/absence (BLAST results, genomic regions covered, and PCR confirmation).

As noted in the manuscript, DENV reads were detected in two distinct genomic regions by metagenomic sequencing, and these findings were further supported by real-time PCR targeting a separate genomic region not included in the metagenomic analysis (Lines 168-174 and 191–195). Together, these results provide strong evidence for DENV circulation in the DRC.

d. I suggest removing the pan-serotype analysis of DENV, as more in-depth analysis with serotype-specific phylogenies would be more accurate and useful.

Response: See responses above - DENV phylogenetic analysis has been removed.

e. I recommend using a threshold of 80 for highlighting nodes in the phylogenetic tree.

Response: We have implemented the suggested threshold and revised all phylogenetic trees accordingly (Figures 6, S4b, S5b, S6b, S7b, S10, and S11).

3. Absence of investigation of insect-specific viruses

The authors mention detecting several species of insect-specific viruses in several mosquito pools. Why don't they attempt consensus building and phylogenetic analysis of these viruses ? Such data could provide indirect elements to better understand exchanges across the different areas analyzed and e.g. help analyze the surprising clustering results of the PCA.

Response: Thank you for this suggestion. In the revised manuscript, we now include new analyses/visualizations for the top five insect-specific viruses. The consensus sequences generated from *Hubei mosquito virus 2* reads were short; so we included the BLAST results in the revised manuscript (Lines 214-218). Coverage plots and phylogenetic analysis results of the consensus sequences of the other four insect-specific viruses are shown in Figures S4-S7. In addition, we have added a paragraph to the Discussion regarding these insect-specific viruses (Lines 302-313).

Minor comments

The introduction is quite brief. Additional context regarding the Kempese region would be useful there.

Response: Thank you for the comment. While word counts limit additions, we added brief contextual information about the Kimpese region in the Introduction (Lines 73–75) as requested.

L54. Please use suitable rather than conducive.

Response: We used the term “suitable” in the revised manuscript (Line 54).

L69-72. Please provide references for this statement.

Response: We have added the following references to support this statement (Line 75).

1. Otshudiema, J. O. *et al.* Yellow fever outbreak - Kongo Central Province, Democratic Republic of the Congo, August 2016. *MMWR Morb. Mortal. Wkly. Rep.* 66, 335–338 (2017).
2. Mbanzulu, *et al.* Mosquito-borne viral diseases in the Democratic Republic of the Congo: a review, Feb 2020, *Parasit Vectors*, 13(1):103. doi: 10.1186/s13071-020-3985-7.

Methods. Why don’t the authors build the tree with iqtree directly following the identification of the best-fit model ?

Response: We considered this approach but opted to use RAxML-NG instead based on benchmarking data by Kozlov et al. (2019) comparing RAxML-NG against RAxML, ExaML, and IQ-TREE using a collection of empirical datasets. They found that RAxML-NG identified the best-scoring tree for the largest number of datasets (19/21) among all programs tested, while running 1.3× to 4.5× faster.

Reference:

Kozlov, A. M., Darriba, D., Flouri, T., Morel, B. & Stamatakis, A. RAxML-NG: a fast, scalable and user-friendly tool for maximum likelihood phylogenetic inference. Bioinformatics 35, 4453–4455 (2019).

L186-188. Please provide a more detailed view of how that should be implemented. As evidenced in the present study, the chances of identifying human/animal pathogens in a given location are not that high, so efforts should be put into describing a suitable sampling/surveillance scheme for these activities.

Response: Ultimately, the choice of approach varies depending on the use case. We have expanded the Discussion to describe suitable sampling and surveillance strategies, including recommendations on spatial and temporal sampling intensity and longitudinal surveillance, to improve the likelihood of detecting rare or sporadically circulating pathogens (Lines 236–249). In addition, because single-timepoint sampling of selected *Aedes* pools may limit the detection of rare or sporadically circulating human or animal pathogenic arboviruses, we have added a sentence acknowledging this limitation in the revised manuscript (Lines 345–347).

L189-190. Any in the Kempese area ? Or close to it ?

Response: We detected DENV by RT-PCR in three of the eight randomly selected mosquito pools. Two of these pools were collected in Kimpese city and one in Viaza (Table S6). Both locations are within the broader Kimpese region, which is why we refer to the "Kimpese region" in Lines 250-251.

L196-198. Not useful here in my opinion as there is no report of severe dengue cases to discuss in that specific context as far as the discussion goes.

Response: We have deleted this sentence in the revised manuscript (Lines 257–260).

L200-202. Concerning, what about available sequences from neighbouring countries ?

Response: Thank you for the comments. The DENV BLAST results indicate that all DENV-4 reads detected in the six positive pools are highly similar to a published sequence from Thailand (Lines 171–174). No closely related sequences were found in the NCBI database from other neighboring countries.

L262-264. Are there important differences between the different locations in terms of elevation, land use patterns, or vegetation ?

Response: Kimpese, Viaza, and Malanga are all located within Kongo Central Province but differ modestly in elevation (approximately 300m above sea level) and landscape context. All three localities are characterized by predominantly rural–agricultural settings; however, Kimpese serves as a more established market and transport hub, whereas Viaza and Malanga represent smaller, village-based agricultural communities within a similar savanna–farmland mosaic. Although not statistically significant, the clustering observed among samples from Kimpese city and Viaza in the PCA suggests potential geographical structuring within local *Aedes* mosquito populations. This text has been added to the manuscript (Lines 329–335).

REVIEWER COMMENTS

We thank the reviewers for their helpful comments. Line numbers used in the point-by-point responses below correspond to the “tracked changes” version of the manuscript.

Reviewer #3 (Remarks to the Author):

I don't understand the sequencing results l 78.

A single sample containing all the pools was sequenced. If that's the case, it should be stated beforehand. Otherwise, if the authors created several sequencing libraries and pooled all the reads, it makes no sense and the analysis will probably have to be redone.

Response: Thanks for the comments. Mosquitos were collected from the field and processed in pools. We generated one sequencing library for each of eight field mosquito pools, as well as a laboratory-uninfected *Ae. aegypti* mosquito pool and a water pool, as controls. Each library was barcoded during library preparation with unique dual indices (NEBNext Multiplex Oligos for Illumina) (Lines 389-392), enabling unambiguous demultiplexing of reads per pool post-sequencing. In the previous version, we reported the total sequencing reads across all libraries. We have added per-pool read counts in **Figure 3a** and Lines 85–93. Using this dual-indexing approach, it is possible to unambiguously demultiplex the data during analysis.

This group is mentioned as l96 "laboratory-uninfected Ae. aegypti mosquito pool", but the group has not been described, including in the mosquito collection description. paragraphaph l96-107 hard to understand, need to be reformulated

Response: The "laboratory-uninfected *Ae. aegypti* mosquito pool" information is detailed in Lines 356–358 and 458–460. The paragraph mentioned by the reviewer describes contamination filtering and viral detection workflow. We have revised this paragraph (Lines 99-113) to clarify the filtering logic and read retention criteria.

l 131: descriptions of kraken or filter conditions should not be included in the text; they should appear before the description of sequencing results.

Response: Thanks for the comment. Filtering conditions are described in the Methods section (Lines 417–422). All main manuscript results reflect post-filtering data. We have retained text in Lines 137–138 to direct readers to the Supplementary Notes for pre-filtering results.

Reviewer #4 (Remarks to the Author):

The authors made several changes to their manuscript following the last round of reviews, addressing most comments. I have only one minor comment remaining (see below). As mentioned in my previous review, this study has value as it is a rare xenosurveillance initiative, covering an understudied region endemic for multiple arboviruses, likely prone to virus emergence, and where sylvatic and urban arbovirus transmission cycles may overlap. But in spite of the interest of this study, the small sample size and the limited genomic coverage still restrict the scope of the results and the conclusions of the study. For this reason, I do no think this study meets the high standards of this journal.

Response: Thank you for the comments. We agree that the small sample size is one of our limitations (Lines 317-320) and that sequencing more pools would increase the generalizability of our findings and allow for more robust region-level comparisons. We also agree that limited DENV genomic coverage is a limitation of our study; this is well described in the manuscript (Lines 320-327). However, additional sequencing could bias results owing to differences in sample age at the time of processing. More extensive sampling is outside the scope of the current manuscript, but is something we are working toward in the future.

We appreciate the reviewer's comment about the value of xenosurveillance efforts in arbovirus-prone and understudied regions like the DRC. Despite acknowledged limitations, multiple lines of evidence give us confidence that our DENV calls are correct. Furthermore, our findings are an important contribution to our understanding of the region's complex arboviral landscape. These results are part of ongoing discussions in the DRC about the need for diversified vector control strategies.

Minor comment:

l220-222 and 307-308: Please provide values to allow the reader to formally assess what is meant by "highly similar"

Response: These values are reported in the Results section (Lines 150-156, 191-194, and 198-199). We have also added new supplementary tables (**Table S9-S12**) with BLAST results for four insect-specific viruses.

REVIEWERS' COMMENTS

We thank the reviewers for their helpful comments. Line numbers used in the point-by-point responses below correspond to the “tracked changes” version of the manuscript.

Reviewer #4 (Remarks to the Author):

The reviewers have addressed all my technical comments in the previous round of reviews. As mentioned in my previous review, in spite of the interest of this xenomonitoring study covering an understudied region endemic for multiple arboviruses, the small sample size and the limited genomic coverage still restrict the scope of the results and the conclusions of the study. For this reason, I do no think this study meets the high standards of Nature Communications.

Response: We thank the reviewer for their thoughtful evaluation and for acknowledging that all technical comments have been satisfactorily addressed in the previous round.

We respectfully disagree with the assessment that the study does not meet the standards of the journal. While we acknowledge that the sample size and genomic coverage are limitations, these constraints are inherent to xenomonitoring studies conducted in understudied and resource-limited regions. Despite these limitations, our findings provide insights into arbovirus circulation in a geographically and epidemiologically important area that remains largely underrepresented in the literature. Strengths of this work include (i) generating rare data from an understudied region endemic for multiple arboviruses, (ii) demonstrating the feasibility and utility of xenomonitoring in this context, and (iii) providing a foundation for future, more extensive genomic surveillance efforts. We maintain our position that the study offers meaningful and timely contributions to the field.

Reviewer #5 (Remarks to the Author):

MEDIATING REFEREE REPORT
NCOMMS-24-84684C
Nature Communications
Manuscript: "Evidence of dengue virus transmission and a diverse Aedes mosquito virome on the Democratic Republic of Congo-Angola border" by Parr et al.

Overall Assessment

The authors have made genuine and meaningful efforts to address Reviewer #3's three specific concerns. The most substantive issue — confusion about the sequencing design — has been fully and clearly resolved. The remaining two concerns have been substantially but not entirely addressed, and I note minor residual issues for each, as detailed below.

Response: Thank you for your comments. Please find our responses below, corresponding to the tracked-changes version of the manuscript.

Comment 1: Confusion About the Sequencing Setup

Original concern: The reviewer was unclear whether a single pooled sample had been sequenced or whether multiple libraries had been generated and their reads combined — and noted that the latter approach would require the analysis to be redone.
Authors' response: The authors clarify that each of the eight field mosquito pools, along with the laboratory-uninfected Ae. aegypti control and the water control, was prepared as an individually barcoded sequencing library using unique dual indices (NEBNext Multiplex Oligos for Illumina). These libraries were sequenced together on a single NovaSeq run and subsequently demultiplexed post-hoc. Per-pool read counts are now clearly presented in the revised Figure 3a (ranging from approximately 360 million to 598 million raw reads per pool) and described in Lines 85-91 of the revised manuscript.

Verdict: This concern is fully and satisfactorily addressed. The sequencing design is now unambiguous and methodologically sound.

Response: Thank you for your comment.

Comment 2: Missing Description of the Laboratory Control; Paragraph Clarity
Original concern: The reviewer noted that the "laboratory-uninfected Ae. aegypti mosquito pool" had not been described in the mosquito collection section, and that the paragraph covering contamination control (formerly Lines 96-107) was hard to understand and needed reformulation.

Authors' response: The laboratory control is now described in the Methods section (Lines 351-353), where the authors state that 20 uninfected Ae. aegypti mosquitoes (strain NR-48920, obtained from BEI Resources via NIH/NIAID) were pooled to serve as a negative control. The formerly problematic paragraph has been revised (Lines 97-109 in the revised manuscript) to more clearly explain: (i) the purpose of the laboratory and water controls; (ii) how residual reads from these controls were assembled into contaminating contigs; (iii) how those contigs were used to filter reads in field mosquito pools; and (iv) why no reads were ultimately removed from field pools,

supported by BLAST evidence showing that overlapping viral genera mapped to distinct genomic regions in field pools compared to controls.

Verdict: This concern is substantially addressed. The description of the laboratory control has been added appropriately, and the logic of the contamination filtering workflow is now considerably clearer. However, the paragraph remains technically dense and may still be challenging for readers unfamiliar with metagenomic workflows. I would encourage the authors to consider one further simplification pass on this paragraph to improve accessibility for a broad readership (if the Editor/Authors share the same feeling).

Response: Thank you for this constructive comment. We made several revisions to this section to streamline the language and improve clarity. We have clarified the step-by-step logic of the contamination filtering workflow to enhance readability for a broad audience (Lines 101-116).

Comment 3: Bioinformatic Filter Descriptions Should Appear in Methods, Not Results
Original concern: The reviewer requested that descriptions of KrakenUniq and filter conditions (RPM threshold, index-hopping filter) be moved from the Results section to the Methods section.
Authors' response: The authors confirm that filtering conditions are now fully described in the Methods section (Lines 417-422), and that the revised Results section includes only a brief pointer (Lines 137-138) directing readers to the Supplementary Notes for pre-filtering results.

Verdict: This concern is partially addressed. While filtering parameters are now appropriately detailed in the Methods, Lines 105-109 of the revised Results section still contain specific filtering threshold details (RPM <10 threshold and index-hopping filter criteria). The authors' justification for retaining this brief mention — to contextualise the reported numbers — is pragmatic and not unreasonable. Although it does not fully honour the reviewer's original intent to remove such methodological descriptions from the Results text, it does no harm as it provides context to readers.

Recommendation
The authors have responded constructively to Reviewer #3's comments and the manuscript has been meaningfully improved. The most critical concern regarding the sequencing design has been fully resolved. I recommend minor revisions to address comment 2: i.e. a further clarity pass on the contamination filtering paragraph (Lines 97-109.

Response: We thank the reviewer for the positive evaluation of our revisions and for acknowledging the improvements to the manuscript. We have addressed the remaining minor comment by further revising the contamination filtering paragraph to improve clarity, readability, and accessibility (Lines 101-116).
