# Supplementary material for: Evidence of dengue transmission and a diverse Aedes mosquito virome on the Congo’s Angola border
Source: Nat Commun. 2026 Aug 10;17:9560. doi: 10.1038/s41467-026-76453-z (PMC13550466; doi:10.1038/s41467-026-76453-z)
Supplement: Supplementary file 3 [file 41467_2026_76453_MOESM3_ESM.pdf]

nature portfolio

Corresponding author(s): Wenqiao He; Jonathan B. Parr

Last updated by author(s): Jun 25, 2026

Reporting Summary

Nature Portfolio wishes to improve the reproducibility of the work that we publish. This form provides structure for consistency and transparency in reporting. For further information on Nature Portfolio policies, see our Editorial Policies and the Editorial Policy Checklist.

Statistics

For all statistical analyses, confirm that the following items are present in the figure legend, table legend, main text, or Methods section.

n/a	Confirmed	
☐	☒	The exact sample size (*n*) for each experimental group/condition, given as a discrete number and unit of measurement
☐	☒	A statement on whether measurements were taken from distinct samples or whether the same sample was measured repeatedly
☒	☐	The statistical test(s) used AND whether they are one- or two-sided<br>*Only common tests should be described solely by name; describe more complex techniques in the Methods section.*
☒	☐	A description of all covariates tested
☒	☐	A description of any assumptions or corrections, such as tests of normality and adjustment for multiple comparisons
☒	☐	A full description of the statistical parameters including central tendency (e.g. means) or other basic estimates (e.g. regression coefficient) AND variation (e.g. standard deviation) or associated estimates of uncertainty (e.g. confidence intervals)
☒	☐	For null hypothesis testing, the test statistic (e.g. *F*, *t*, *r*) with confidence intervals, effect sizes, degrees of freedom and *P* value noted<br>*Give P values as exact values whenever suitable.*
☒	☐	For Bayesian analysis, information on the choice of priors and Markov chain Monte Carlo settings
☒	☐	For hierarchical and complex designs, identification of the appropriate level for tests and full reporting of outcomes
☒	☐	Estimates of effect sizes (e.g. Cohen's *d*, Pearson's *r*), indicating how they were calculated

Our web collection on statistics for biologists contains articles on many of the points above.

Software and code

Policy information about availability of computer code

Data collection	NA
Data analysis	Analysis code is publicly available at https://github.com/IDEELResearch/DRCAedesSeq. The following softwares and packages are used for data analysis: BBMerge (version 38.96), Trimmomatic (version 0.36), bwa-mem2 (version 2.2.1), SAMtools (version 1.21), metaSPAdes (version 4.0.0), KrakenUniq (version 1.0.4), BLAST (version 2.14.1), metaSPAdes (version 4.0.0), MAFFT (version 7.490), RAxML-NG (version 1.2.2), IQ-TREE (version 2.4.0), Guppy (version 5.6.7), R software (version 4.2.0), RStudio (version 2022.02.2), ggrepel (version 0.9.6), ggplot2 (version 3.5.2), tidyverse (version 2.0.0), eulerr (version 7.0.2), viridis (version 0.6.5), scico (version 1.5.0), ape (version 5.8-1), ggtree (version 3.16.0), rentrez (version 1.2.4), countrycode (version 1.6.1), RColorBrewer (version 1.1-3), dplyr (version 1.1.4), sf (version 1.0.21), rnaturalearth (version 1.0.1), and rnaturalearthdata (version 1.0.0).

For manuscripts utilizing custom algorithms or software that are central to the research but not yet described in published literature, software must be made available to editors and reviewers. We strongly encourage code deposition in a community repository (e.g. GitHub). See the Nature Portfolio guidelines for submitting code & software for further information.

Data

Policy information about availability of data

All manuscripts must include a data availability statement. This statement should provide the following information, where applicable:

- Accession codes, unique identifiers, or web links for publicly available datasets
- A description of any restrictions on data availability
- For clinical datasets or third party data, please ensure that the statement adheres to our policy

The NovaSeq and MinION sequencing data generated in this study have been deposited in the NCBI Sequence Read Archive database under accession codes PRJNA1200724 [PRJNA1200724 - SRA - NCBI] and PRJNA1200731 [PRJNA1200731 - SRA - NCBI]. This study utilized publicly available sequence datasets from NCBI Virus, RefSeq, as well as GenBank databases [https://www.ncbi.nlm.nih.gov/]. Accession numbers for publicly available sequences used in this study are provided in Supplementary Tables 16-22.

Research involving human participants, their data, or biological material

Policy information about studies with human participants or human data. See also policy information about sex, gender (identity/presentation), and sexual orientation and race, ethnicity and racism.

Reporting on sex and gender	No human participants included in this study.
Reporting on race, ethnicity, or other socially relevant groupings	No human participants included in this study.
Population characteristics	No human participants included in this study.
Recruitment	No human participants included in this study.
Ethics oversight	No human participants included in this study.

Note that full information on the approval of the study protocol must also be provided in the manuscript.

Field-specific reporting

Please select the one below that is the best fit for your research. If you are not sure, read the appropriate sections before making your selection.

☐ Life sciences ☐ Behavioural & social sciences ☒ Ecological, evolutionary & environmental sciences

For a reference copy of the document with all sections, see nature.com/documents/nr-reporting-summary-flat.pdf

Ecological, evolutionary & environmental sciences study design

All studies must disclose on these points even when the disclosure is negative.

Study description	Aedes mosquitoes are particularly important vectors for multiple important human pathogens, including dengue virus (DENV), Zika virus (ZIKV), yellow fever virus (YFV), and chikungunya virus (CHIKV). A recent survey found that Aedes albopictus is expanding its distribution in the DRC and displacing native Aedes species. This study using field-collected Aedes mosquitoes from the Democratic Republic of Congo (DRC). We collected Aedes mosquitoes in the DRC and used metagenomic and targeted sequencing to investigate their virome, species, and blood meals.
Research sample	Adult Aedes mosquitoes were collected from three health areas in Kimpese, DRC, and then pooled according to sample collection site (Kimpese city, Malanga, and Viaza). The final mosquito pools were randomly selected from mosquito pools collected across three health areas in the DRC (see Figure 2). These pools represent approximately 20% of the total mosquito pools from each region. The selected mosquito pools were washed with 70% ethanol and rinsed twice with molecular-grade water before removal of mosquito heads. Total RNA was extracted from mosquito bodies from eight selected field mosquito pools, then underwent viral metagenomic sequencing, targeted sequencing, and real-time PCR.
Sampling strategy	This is an observational study into virome in Aedes mosquitoes in the DRC. No formal sample size calculation was performed; however, the number of samples was determined based on field collection feasibility and was sufficient to capture viral diversity across locations. The final mosquito pools were randomly selected to represent approximately 20% of the total mosquito pools from each region. Differences in the number of mosquito pools among health areas largely represent variations in the quantity of mosquito pools collected in each area.
Data collection	Mosquitoes were collected in three health areas in Kimpese, DRC, Ceco (Kimpese city), Malanga, and Viaza by T.B. and F.V. All collections were performed outdoors, and GPS coordinates along with a brief description of collection sites were recorded by pen and paper. Collected mosquitoes were morphologically identified to the species level following the Huang morphological key. Aedes species were pooled by sample collection site. All field mosquito pools were preserved in DNA/RNA Shield (ZYMO research, CA, USA), shipped to the University of North Carolina at Chapel Hill (UNC), and stored at -80°C prior to additional screening, processing, and analysis (see "Mosquito collection").

Timing and spatial scale	Mosquitoes were collected from April 23 - 30, 2022, during the rainy season, when mosquito density is high. All collections were performed outdoors in three health areas in Kimpese, DRC, Ceco (Kimpese city), Malanga, and Viaza (see Figure 2a).
Data exclusions	Collected mosquitoes were morphologically identified to the species level following the Huang morphological key. Targeted sequencing was performed to confirm the mosquito species. Only Aedes mosquitoes were included in this study.
Reproducibility	This observational study into virome in Aedes mosquitoes in the DRC did not have an experimental component and could not be replicated within the scope of the study.
Randomization	Eight field Aedes mosquito pools were randomly selected from the three health areas: five from Kimpese city, one from Malanga, and two from Viaza (see Figure 2b). These pools represent approximately 20% of the total mosquito pools from each region.
Blinding	Blinding was not relevant because this was not an experimental study.

Did the study involve field work? ☒ Yes ☐ No

Field work, collection and transport

Field conditions	Mosquito collection were performed over a 7-day period. All collections were performed outdoors. Mosquitoes were collected from 3 to 6 pm. Electric Aspirator (Prokopack Aspirator, John W. Hock, Gainesville, USA) and BG sentinel traps (Biogents Inc, Regensburg, Germany) baited with dry ice were used to collect mosquitoes.
Location	All collections were performed in three health areas in Kimpese, DRC, Ceco (Kimpese city), Malanga, and Viaza (see Figure 2a).
Access & import/export	Samples were exported from their collection sites in the DRC in accordance with the policies of local governments and institutional collaborators in the country.
Disturbance	The mosquito collection did not impose any significant disturbance on local populations.

Reporting for specific materials, systems and methods

We require information from authors about some types of materials, experimental systems and methods used in many studies. Here, indicate whether each material, system or method listed is relevant to your study. If you are not sure if a list item applies to your research, read the appropriate section before selecting a response.

Materials & experimental systems

n/a	Involved in the study	
☒	☐	Antibodies
☒	☐	Eukaryotic cell lines
☒	☐	Palaeontology and archaeology
☐	☒	Animals and other organisms
☒	☐	Clinical data
☒	☐	Dual use research of concern
☒	☐	Plants

Methods

n/a	Involved in the study	
☒	☐	ChIP-seq
☒	☐	Flow cytometry
☒	☐	MRI-based neuroimaging

Animals and other research organisms

Policy information about studies involving animals; ARRIVE guidelines recommended for reporting animal research, and Sex and Gender in Research

Laboratory animals	The laboratory un-infected Aedes aegypti mosquitoes were provided by the NIH/NIAID Filariasis Research Reagent Resource Center for distribution through BEI Resources, NIAID, NIH: Uninfected Aedes aegypti , Strain Black Eye Liverpool (Frozen), NR-48920.
Wild animals	Adult Aedes mosquitoes were collected as described above.Collected mosquitoes were morphologically identified to the species level following the Huang morphological key and then pooled according to sample collection sites. All mosquito pools were preserved in DNA/RNA Shield and stored at -80°C prior to shipment to UNC for additional screening, processing, and analysis (see "Mosquito collection"). The selected mosquito pools were washed with 70% ethanol and rinsed twice with molecular-grade water before removal of mosquito heads. Total RNA was extracted and used for viral metagenomic sequencing, targeted sequencing, and real-time PCR.
Reporting on sex	Mosquitoes were not sexually-differentiated during this study.
Field-collected samples	Adult Aedes mosquitoes were collected and processed as described above.
Ethics oversight	US and DRC IRB approvals are not required for studies of invertebrates.

Note that full information on the approval of the study protocol must also be provided in the manuscript.

Plants

Seed stocks	No plants were involved in this study.
Novel plant genotypes	No plants were involved in this study.
Authentication	No plants were involved in this study.
